# Osteopontin associates with brain T_RM_-cell transcriptome and compartmentalization in donors with and without multiple sclerosis

**DOI:** 10.1016/j.isci.2022.105785

**Published:** 2022-12-09

**Authors:** Cheng-Chih Hsiao, Hendrik J. Engelenburg, Aldo Jongejan, Jing Zhu, Baohong Zhang, Michael Mingueneau, Perry D. Moerland, Inge Huitinga, Joost Smolders, Jörg Hamann

**Affiliations:** 1 Neuroimmunology Research Group, Netherlands Institute for Neuroscience, 1105 BA Amsterdam, the Netherlands; 2 Department of Experimental Immunology, Amsterdam Institute for Infection and Immunity, Amsterdam University Medical Centers, 1105 AZ Amsterdam, the Netherlands; 3 Bioinformatics Laboratory, Department of Epidemiology and Data Science, Amsterdam University Medical Centers, 1105 AZ Amsterdam, the Netherlands; 4 Translational Biology, Biogen, Cambridge, MA 02142, USA; 5 Multiple Sclerosis and Neurorepair Research Unit, Biogen, Cambridge, MA 02142, USA; 6 Swammerdam Institute for Life Sciences, Center for Neuroscience, University of Amsterdam, 1098 XH Amsterdam, the Netherlands; 7 MS Center ErasMS, Departments of Neurology and Immunology, Erasmus Medical Center, 3015 GD Rotterdam, the Netherlands

**Keywords:** Neuroscience, Molecular neuroscience, Omics, Transcriptomics

## Abstract

The human brain is populated by perivascular T cells with a tissue-resident memory T (T_RM_)-cell phenotype, which in multiple sclerosis (MS) associate with lesions. We investigated the transcriptional and functional profile of freshly isolated T cells from white and gray matter. RNA sequencing of CD8^+^ and CD4^+^ CD69^+^ T cells revealed T_RM_-cell signatures. Notably, gene expression hardly differed between lesional and normal-appearing white matter T cells in MS brains. Genes up-regulated in brain T_RM_ cells were *MS4A1* (CD20) and *SPP1* (osteopontin, OPN). OPN is also abundantly expressed by microglia and has been shown to inhibit T cell activity. In line with their parenchymal localization and the increased presence of OPN in active MS lesions, we noticed a reduced production of inflammatory cytokines IL-2, TNF, and IFNγ by lesion-derived CD8^+^ and CD4^+^ T cells *ex vivo*. Our study reports traits of brain T_RM_ cells and reveals their tight control in MS lesions.

## Highlights

• Human brain T cells possess a T_RM_-cell phenotypic and transcriptional profile
• Human brain T_RM_ cells are characterized by CD20 and osteopontin expression
• In MS lesional T cells, the brain T_RM_-cell transcriptome is preserved
• MS lesional T_RM_ cells produce less inflammatory cytokines on stimulation

## Graphical abstract

**Figure:**
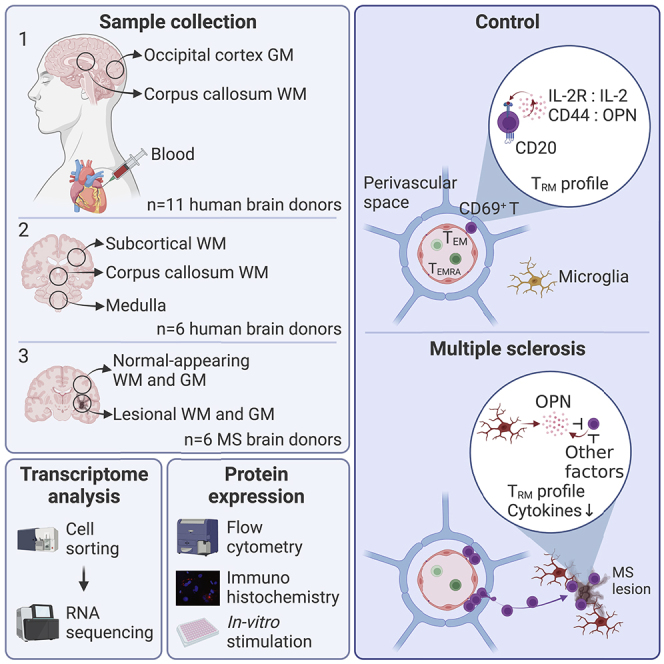

## Introduction

The human central nervous system (CNS) is an immune-privileged site with a tight control of inflammatory responses.^1^^,^^2^ This status has been attributed to a combination of strictly compartmentalized organization, regulated transportation of molecules over the blood–brain barrier, parenchymal expression of immune-regulatory molecules, and controlled trafficking of surveilling immune cells. The CNS is surrounded by meninges containing the subarachnoid space, where cerebrospinal fluid (CSF) circulates. In individuals without brain diseases, this compartment is patrolled by more CD4^+^ than CD8^+^ T cells with a most dominant CCR7^+^ central memory T cell (T_CM_) phenotype.^3^^,^^4^ Choroid plexus and meningeal vasculature have been identified as main ports of entry for T cells in the subarachnoid space,^4^^,^^5^ with specialized meningeal lymphoid vessels mediating return toward secondary lymphoid organs.^6^

In predominantly postmortem studies, the white matter (WM) areas of the brain have been shown to contain low numbers of T cells.^7^ These cells are primarily located in the perivascular space (PVS) surrounding the infiltrating and draining vessels of WM.^8^ Although this compartment is a continuum of the subarachnoid space, phenotypically different T cells have been noted. The PVS contains more CD8^+^ compared to CD4^+^ T cells, which are characterized by a dominant CCR7^−^ CD69^+^CD103^+/−^ tissue-resident memory T (T_RM_)-cell phenotype.^2^^,^^8^^,^^9^ Persisting in various human tissues,^10^ T_RM_ cells are believed to be remnants of a prior primary antigen encounter facilitating a rapid local recall response after re-challenge with their antigen. Accordingly, in animal studies, infections with neurotropic viruses drive development of T_RM_-cell pools in the brain.^11^^,^^12^ Lack of re-circulation molecules on T_RM_ cells causes their permanent retention to specific tissue compartments.^13^ However, the strict localization of brain T_RM_ cells in the PVS contrasts to the more scattered distribution found in other tissues.^14^ In addition, we observed low expression of CD20 to mark subsets of human brain T_RM_ cells,^15^ which has not been reported as a prominent feature in other tissues. These observations suggest the presence of unique features of brain T_RM_ cells adapting to residence within the unique CNS environment.

Brain T_RM_ cells may not only be innocent sentinels but also contribute detrimentally to neuroinflammatory diseases. In late-stage multiple sclerosis (MS) autopsies, we and others showed that brain T cells expand and localize in the brain parenchyma in association with active MS lesions.^16^^,^^17^ MS lesional T cells showed expression of core T_RM_-cell markers (CD69, CD103, CD49a, PD-1, and CD20), although differential expression of some markers suggested a higher migratory potential (CXCR6), a higher activation state (Ki-67), and a higher cytotoxic potential (GPR56).^15^^,^^17^ Intriguingly, a clonal expansion of CSF CD8^+^ T cells displaying T_RM_-cell like characteristics has been noted in MS-discordant twins and has been argued to be one of the earliest events in the onset of MS.^18^ How brain T_RM_ cells are recruited and if and how these cells contribute to ongoing disease activity in advanced MS remains to be investigated.

The aim of our current study was to further consolidate the phenotype of human brain T_RM_ cells and to identify dominant or unique features relevant for their local compartmentalization and control. Furthermore, we explored the versatility of this profile in association with lesion formation in MS.

## Results

### Brain CD69^+^ T cells possess a T_RM_-cell transcriptomic profile

**Figure 1:**
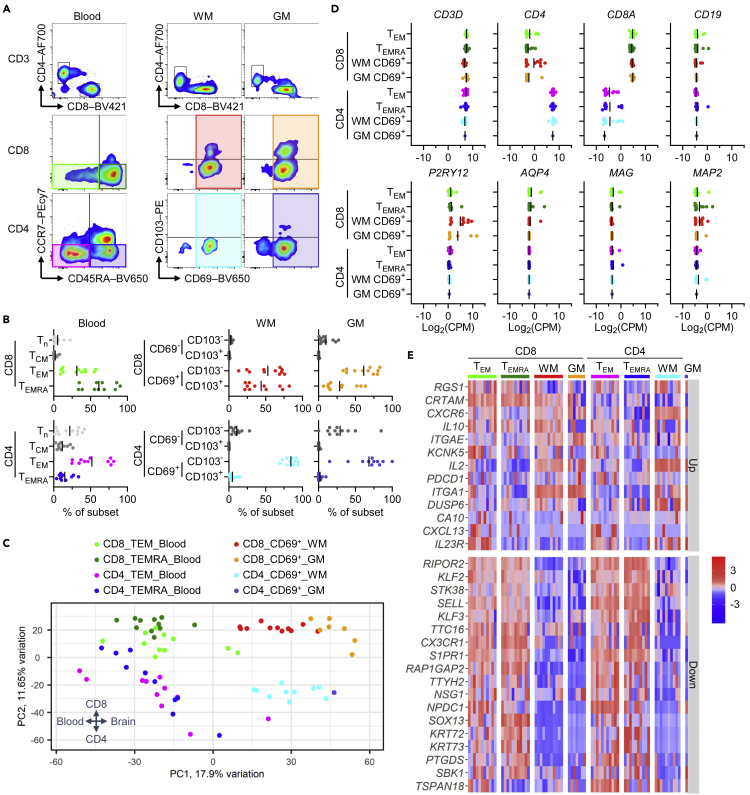
Whole-transcriptome profiling of brain CD8^+^and CD4^+^ CD69^+^ T cells Bulk RNA sequencing was performed of corresponding CD8^+^ and CD4^+^ blood T_EM_ and T_EMRA_ cells and brain WM and GM CD69^+^ T cells from n = 11 brain donors (first dataset). (A) Representative plots showing the flow cytometry gating strategy used for obtaining the samples for RNA sequencing. (B) Percentage of CD8^+^ and CD4^+^ T cell subsets in the blood and brain samples processed for RNA sequencing, determined by flow cytometry. (C) Expression of genes associated with T cells (*CD3D*, *CD4*, *CD8A*), B cells (*CD19*), microglia (*P2RY12*), astrocytes (*AQP4*), oligodendrocytes (*MAG*), and neurons (*MAP2*) in the sequenced samples, obtained by RNA sequencing. CPM, counts per million. (D) PCA of gene expression in the analyzed samples. (E) Heatmap showing row-scaled expression levels of T_RM_-cell core signature genes in corresponding CD8^+^and CD4^+^ blood T_EM_ and T_EMRA_ cells and brain WM and GM CD69^+^ T cells. T_n_, naive T cells. Up/down labels relate to their directionality in the T_RM_-cell core signature gene set. Gene expression was corrected for cell number.

CD8^+^ and CD4^+^ T cells of corpus callosum WM, cortical gray matter (GM), and paired peripheral blood were isolated from n = 11 deceased human brain donors that subsequently came to obduction at the Netherlands Brain Bank (Figure S1). Blood cells were sorted into effector memory T cells (T_EM_) and effector memory T cells re-expressing CD45RA (T_EMRA_) based on co-expression of CCR7 and CD45RA (Figure 1A). Brain cells were sorted for presence of the T_RM_-cell marker CD69, found on 96.9 ± 1.4% of WM CD8^+^ T cells, 89.9 ± 9.2% of GM CD8^+^ T cells, 89.1 ± 7.2% of WM CD4^+^ T cells, and 70.4 ± 22.9% of GM CD4^+^ T cells. As expected, 43.9 ± 22.2% of WM CD8^+^ T cells and 28.6 ± 20.3% of GM CD8^+^ T cells, but almost no WM or GM CD4^+^ T cells expressed CD103 (Figure 1B). Brain CD8^+^ T cells showed a strong correlation of CD69^+^CD103^-^ and CD69^+^CD103^+^ proportions between WM and GM (Figure S2). The obtained cell numbers enabled bulk RNA sequencing of all individual blood and brain WM samples, whereas n = 5 CD8^+^ and all CD4^+^ GM samples with cell numbers <2,000 had to be pooled for sufficient input. Principal component analysis (PCA) revealed a clear separation of CD8^+^ and CD4^+^ as well as of blood and brain T cell samples, respectively (Figure 1C). Robust presence of T cell-specific transcripts (*CD3D*, *CD4*, *CD8A*) and low detection of transcripts associated with B cells (*CD19*), microglia (*P2RY12*), astrocytes (*AQP4*), oligodendrocytes (*MAG*), and neurons (*MAP2*) confirmed the purity of the obtained T cell transcriptomes (Figure 1D).

We next compared the obtained transcriptomes with the core gene signature of human CD69^+^ T_RM_ cells reported by Kumar et al., comprising 13 upregulated and 18 downregulated genes.^13^ Brain CD69^+^ T cells matched this signature, indicated by high gene expression of the adhesion molecules *ITGA1* (CD49a) and *ITGAE* (CD103), the interleukins *IL2* and *IL10*, the chemokine receptor *CXCR6*, the dual-specificity phosphatase *DUSP6*, and the T cell-inhibitory receptor *PDCD1* (PD-1) as well as by low gene expression of the lymph node-homing factor *SELL* (CD62L), the Krüppel-like transcription factors *KLF2* and *KLF3*, the sphingosine receptor and immune modulator *S1PR1*, and the fractalkine receptor *CX3CR1*, in comparison to blood T_EM(RA)_ cells (Figure 1E). This was further confirmed in a competitive gene set enrichment analysis (Figure S3). Despite the variable background of the brain donors, consisting of patients with Alzheimer’s disease, Parkinson’s disease, dementia, depression, and MS, as well as controls with no known neurological disorders, brain CD69^+^ memory T cells displayed a consistent transcriptional profile that clearly differed from circulating T_EM(RA)_ cells.

### Brain CD69^+^ T_RM_ cells abundantly express CD20 and OPN

**Figure 2:**
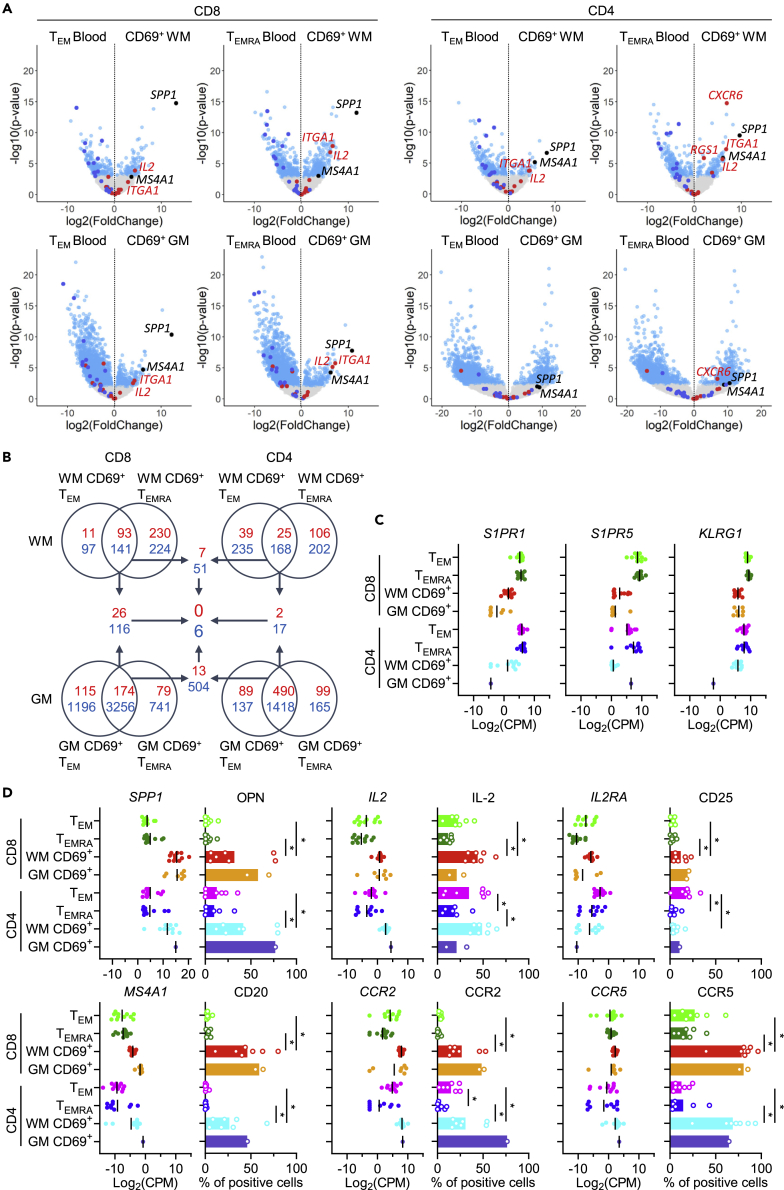
Brain-specific characteristics of CD8^+^ and CD4^+^ CD69^+^ T cells (A) Volcano plots displaying differential gene expression between CD8^+^ and CD4^+^ blood T_EM_ and T_EMRA_ cells and brain WM and GM CD69^+^ T cells. Core signature genes up- and downregulated in T_RM_ cells^13^ are depicted in red and blue, respectively. Further genes of particular interest (*SPP1*/OPN and *MS4A1*/CD20) are shown in black. Light blue points are FDR <0.05. (B) Venn diagram showing DE-gene overlap between CD8^+^ and CD4^+^ blood T_EM_ and T_EMRA_ cells and brain WM and GM CD69^+^ T cells. Arrows indicate further overlap between Venn diagrams. Up- and downregulated genes are depicted in red and blue, respectively. (C) Gene expression of selected genes, consistently downregulated in brain CD69^+^ T cells. (D) Gene and protein expression, the latter obtained by flow cytometry of n = 7 brain donors, of selected DE genes in CD8^+^ and CD4^+^ blood T_EM_ and T_EMRA_ cells and brain WM and GM CD69^+^ T cells. Mann-Whitney U test was used, and p values are shown in the plots. Gene expression was corrected for cell number. CPM, counts per million; ∗p*<0.05*.

Comparison of CD8^+^ and CD4^+^ T cells from WM, GM, and blood disclosed genes expressed at lower or higher levels in brain CD69^+^ T cells (Figure 2A). These differentially expressed (DE) genes comprised generic core signature genes of human T_RM_ cells (see above) as well as genes specifically regulated in brain-resident T cells. Several of these genes were robustly regulated across brain CD69^+^ T cells (Figure 2B and Data S1). Genes consistently underexpressed were *S1PR1*, *S1PR5*, and *KLRG1* (Figure 2C). Overexpressed genes, confirmed at protein level by flow cytometry, were *SPP1*, *IL2*, *IL2RA*, *MS4A1*, *CCR2*, and *CCR5* (Figures 2D and S4). Two genes aroused particular interest. *SPP1* (osteopontin, OPN) was the most upregulated gene in WM and GM CD8^+^ T cells as well as WM CD4^+^ T cells. *MS4A1* (CD20) was upregulated in CD8^+^ WM, CD8^+^ GM, and CD4^+^ WM versus circulating T_EMRA_ cells, upregulated in CD8^+^ GM and CD4^+^ WM and approached significance in CD8^+^ WM T cells versus circulating T_EM_ cells (adjusted p = 0.07), with substantial expression levels (44^th^ percentile of whole transcriptome) (Figure S5). This is in line with the intermediate expression of CD20 by a higher proportion of brain CD8^+^ CD69^+^ T cells compared to circulating CD8^+^ T cells reported recently by us.^15^

**Figure 3:**
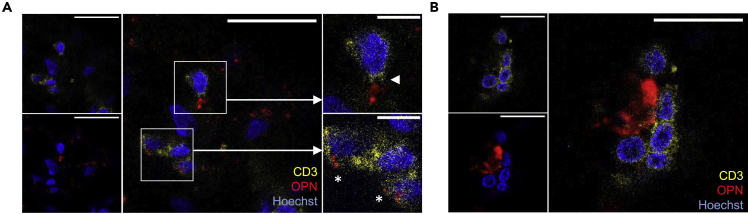
OPN is a spatial hallmark in proximity of brain T_RM_ cells (A) OPN expressed in smaller extracellular deposits in the proximity of T cells (arrowhead) as well as within a T cell (asterisk). (B) T cell aggregation in vicinity of a large extracellular OPN deposition in a non-demented control donor. Shown are immunofluorescence stainings at 63× magnification; scale bar = 20 μm or 5 μm (magnifications).

Given the particularly high gene activity of *SPP1*, we further explored OPN protein expression by brain T cells using immunofluorescence staining. T cells are known to actively secrete OPN.^19^ Accordingly, OPN was observed in extracellular deposits near perivascular but also near the incidental parenchymal T cells (Figure 3A). Frequently, T cells were found in the vicinity of larger aggregates of OPN (Figure 3B). In line with the flow-cytometric analyses, OPN was also detected inside cells, and we therefore quantified extra- and intracellular expression. We noticed that OPN was present in a mean of 75 ± 23% of all T cells (Figure S6). In summary, expression of OPN, together with intermediate presence of CD20, is a hallmark of brain-resident CD69^+^ T cells.

### T_RM_ cells from different brain region possess a similar transcriptome

**Figure 4:**
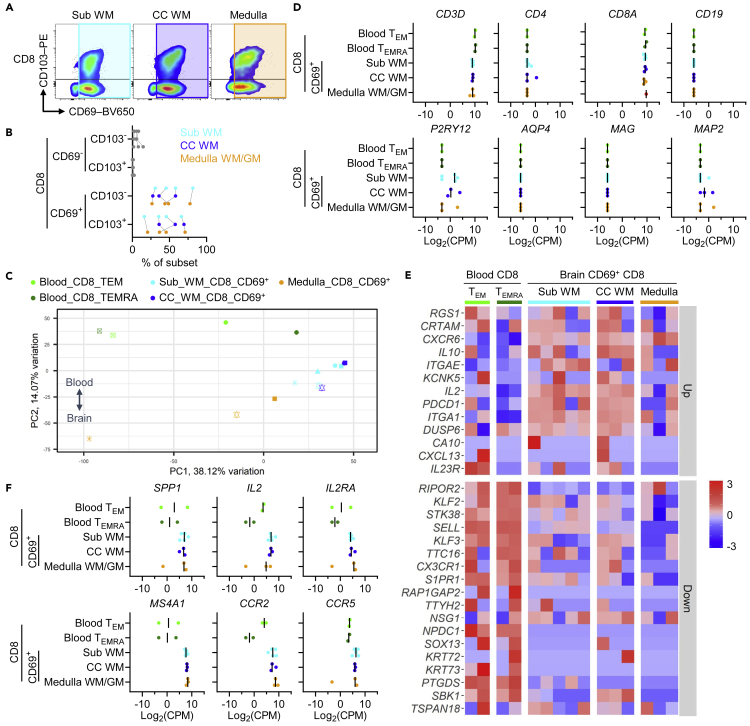
Brain-regional characteristics of CD8^+^ CD69^+^ T cells Bulk RNA sequencing was performed of CD8^+^ CD69^+^ T cells from subcortical WM, corpus callosum WM, and medulla WM/GM from n = 3–5 brain donors (second dataset). (A) Representative dot plots showing the flow cytometry gating strategy used for obtaining the samples for RNA sequencing. (B) Percentage of CD8^+^ T cell subsets in the brain samples processed for RNA sequencing, determined by flow cytometry. (C) Expression of genes associated with T cells (*CD3D*, *CD4*, *CD8A*), B cells (*CD19*), microglia (*P2RY12*), astrocytes (*AQP4*), oligodendrocytes (*MAG*), and neurons (*MAP2*) in the sequenced samples, obtained by RNA sequencing. CPM, counts per million. (D) PCA of gene expression in the analyzed samples. Different symbols represent samples from the same donors. (E) Heatmap showing row-scaled expression levels of T_RM_-cell core signature genes^13^ in corresponding CD8^+^ blood T_EM_ and T_EMRA_ cells and brain CD69^+^ T cells. Gene expression was corrected for cell number. Up/down labels relate to their directionality in the T_RM_-cell core signature gene set. (F) Gene expression of selected T_RM_-cell DE genes in CD8^+^ blood T_EM_ and T_EMRA_ cells and CD69^+^ T cells from different brain regions. CC, corpus callosum; CPM, counts per million; Sub, subcortical.

Previous studies of brain T cells analyzed cells from different WM regions.^8^^,^^9^ To explore regional differences, we isolated CD8^+^ T cells from subcortical WM, corpus callosum WM, and medulla WM/GM of n = 3–5 deceased human brain donors and processed sorted CD69^+^ CD8^+^ T cells for bulk RNA sequencing (Figure 4A). The percentage of all CD8^+^ T cells co-expressing CD69 and CD103 was remarkably similar between brain locations within individual donors (Figure 4B). PCA revealed a clear separation of blood and brain T cell samples as well as a tight clustering of subcortical WM and corpus callosum WM samples, in particular within donors, and, to a lesser extent, medulla samples, representing WM and GM (Figure 4C). The purity of the isolated cells was high (Figure 4D). CD69^+^ T cells from all three brain locations presented a T_RM_-cell core signature^13^ gene expression (Figures 4E and S7, Data S2) and expressed genes abundantly transcribed by brain T_RM_ cells (*SPP1*, *IL2*, *IL2RA*, *MS4A1*, *CCR2*, and *CCR5*) at comparable levels (Figure 4F). There were relatively little DE genes, but WM versus medulla WM/GM samples showed most differences (Data S3). Our data indicate a high similarity between T_RM_ cells in different brain regions and indicate that differences in maturity (CD103 expression) are directed by donor history rather than brain location.

### MS lesion-associated T cells retain a T_RM_-cell signature

**Figure 5:**
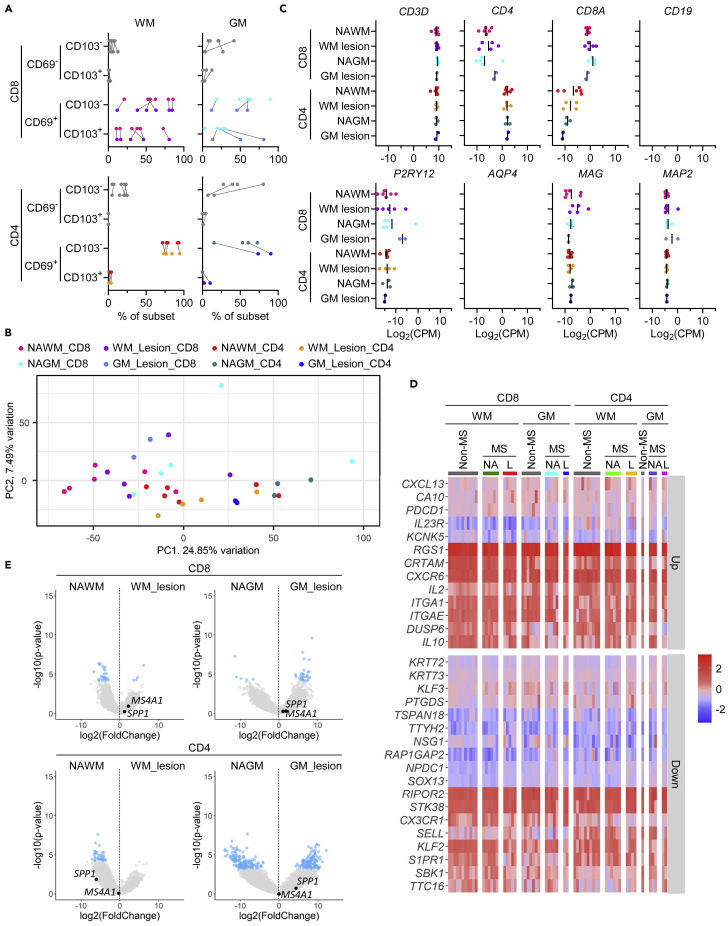
Whole-transcriptome profiling of MS brain CD8^+^ and CD4^+^ CD69^+^ T cells Bulk RNA sequencing was performed of CD8^+^ and CD4^+^ CD69^+^ T cells from MS normal-appearing and lesional WM and GM from n = 6 brain donors (third dataset). (A) Percentage of CD8^+^ and CD4^+^ T cell subsets in the brain samples processed for RNA sequencing, determined by flow cytometry. (B) Expression of genes associated with T cells (*CD3D*, *CD4*, *CD8A*), B cells (*CD19*), microglia (*P2RY12*), astrocytes (*AQP4*), oligodendrocytes (*MAG*), and neurons (*MAP2*) in the sequenced samples, obtained by RNA sequencing. CPM, counts per million. (C) PCA of gene expression in the analyzed samples. (D) Heatmap showing column-scaled expression levels of T_RM_-cell core signature genes^13^ in CD8^+^ and CD4^+^ WM and GM CD69^+^ T cells from non-MS brains (first dataset) as well as from normal-appearing (NA) and lesional (L) tissue of MS brains (third dataset). Gene expression was corrected for cell number and donor. Up/down labels relate to their directionality in the T_RM_-cell core signature gene set. (E) Volcano plots displaying differential gene expression between CD8^+^ and CD4^+^ WM and GM CD69^+^ T cells from normal-appearing and lesional tissue of MS brains. Genes markedly expressed in brain T_RM_ cells are displayed in black. Light blue points are FDR <0.05. NAGM, normal-appearing GM; NAWM, normal-appearing WM.

T cells are implicated in the pathogenesis of MS, and we therefore isolated CD8^+^ and CD4^+^ T cells from normal-appearing and lesional WM and GM of n = 6 deceased MS brain donors (Figure S8). CD69^+^ T cells were sorted and enabled bulk RNA sequencing of normal-appearing WM samples of all 6 donors and normal-appearing GM samples of 2 donors. T cell yield was generally higher in WM compared to GM. MS WM lesions contained n = 5 mixed active/inactive lesions and n = 1 inactive lesion. Again, about half of all CD8^+^ T cells but almost no CD4^+^ T cells in WM expressed CD103 (Figure 5A). PCA revealed clustering of all samples independent of their origin from MS lesional or normal-appearing WM and GM (Figure 5B), and the purity of the obtained T cell transcriptomes was confirmed (Figure 5C). In contrast, as in the non-MS samples, CD8^+^ and CD4^+^ T cells separated quite well.

In a combined PCA of both datasets, we found co-clustering of all brain CD69^+^ T cells suggesting their similarity (Figure S9). In a comparison of T_RM_-cell core signature^13^ gene expression scaled within donors (in columns) across both experiments, CD8^+^ and CD4^+^ CD69^+^ T cells from MS WM and GM lesions were highly similar to cells from MS normal-appearing or non-MS WM and GM (Figure 5D). Differential gene expression analysis revealed a small number of DE genes in MS lesional and normal-appearing WM CD8^+^ and CD4^+^ CD69^+^ T cells (Figure 5E and Data S4).

Because genes associated with MS risk alleles have been postulated to reflect relevant biological pathways in the disease process of MS, which may be reflected in the T_RM_-cell expression profile, we also investigated their expression. There was no enrichment of genes associated with MS risk alleles^20^ in T cells from MS lesions compared to MS normal-appearing WM (Figure S10A and Data S5). There was also no enrichment of MS risk-associated gene expression in T_EM(RA)_ or T_RM_ cells, indicating that the T cell mediated risk of MS onset is not conveyed exclusively by any specific individual T cell subset (Figure S10B and Data S6). Functional enrichment was conversely present. Competitive gene set enrichment analysis (CAMERA) gene set enrichment analysis showed MS lesional WM CD8^+^ CD69^+^ T cells enriched ‘response to virus’, ‘tricarboxylic acid (TCA) cycle’, ‘post-translational modifications’, and ‘transcription/translation’ gene sets, whereas normal-appearing WM CD8^+^ CD69^+^ T cells displayed a trend of ‘acute inflammatory response to antigenic stimulus’ and ‘epithelial cell-cell adhesion’ gene sets (Figure S10C and Data S6). This observation could indicate an altered activation state of lesional T cells.

### MS lesional T cells produce less cytokines *ex vivo* without signs of exhaustion

**Figure 6:**
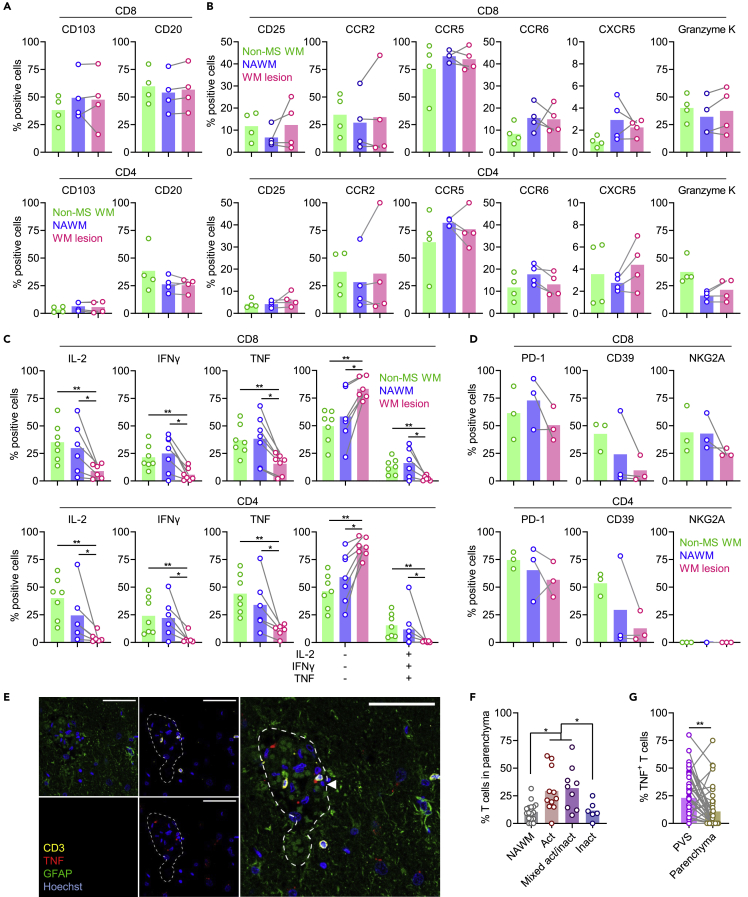
Functional characteristics of MS brain CD8^+^ and CD4^+^ CD69^+^ T cells (A) Percentage of CD8^+^ and CD4^+^ T cells isolated from non-MS WM and MS normal-appearing and lesional WM expressing CD103 and CD20, determined by flow cytometry. (B) Percentage of CD8^+^ and CD4^+^ T cells isolated from non-MS WM and MS normal-appearing and lesional WM expressing the T_H_1/17-associated molecules CD25, CCR2, CCR5, CCR6, CXCR5, and granzyme K, determined by flow cytometry. (C) Cytokine production by CD8^+^ and CD4^+^ T cells isolated from non-MS WM and MS normal-appearing and lesional WM and stimulated for 4 h with PMA/ionomycin, determined by intracellular flow cytometry. Mann-Whitney U test and Wilcoxon matched-pairs signed rank test were used, and p values are shown in the plots. (D) Percentage of CD8^+^ and CD4^+^ T cells isolated from non-MS WM and MS normal-appearing and lesional WM expressing the inhibitory receptors PD-1, CD39, and NKG2A, determined by flow cytometry. (E) Representative immunofluorescence staining image of CD3/TNF/GFAP triple staining. The dotted line indicates the PVS threshold as delineated by GFAP staining. The arrowhead indicates a T cell classified as TNF^+^. 40× magnification, scale bar = 50 μm . (F) Percentage of T cells in the parenchyma of MS tissue, determined by immunofluorescence staining. (G) Percentage of TNF-producing T cells in the PVS and parenchyma of MS NAWM and lesional tissue, determined by immunofluorescence staining. In (F) and (G), pairwise comparisons using estimated marginal means were used, and p values are shown in the plots. ∗p<0.05; ∗∗p<0.01.

Although gene expression did not change at bulk level, functionally relevant subsets may have expanded or decreased in MS lesional compared to normal-appearing WM CD8^+^ and CD4^+^ CD69^+^ T cells (Figure S11). CD103 presence on brain CD8^+^ T cells correlates with reduced expression of differentiation markers, increased expression of tissue-homing chemokine receptors, increased expression of inhibitory receptors PD-1 and CTLA-4, and lower expression of CD20 and the cytolytic granzymes B and K.^8^^,^^17^ Conversely, intermediate presence of CD20 associates with higher expression of chemokine receptors and higher expression of granzymes B and K.^15^ The percentage of CD103^+^ and CD20^+^ brain T cells, measured by flow cytometry, did not differ between MS lesional and normal-appearing WM CD8^+^ and CD4^+^ CD69^+^ T cells (Figures 5A and 6A). In addition, expression of OPN, CD25, granzyme K, chemokine receptors, and inhibitory receptors on CD8^+^ CD69^+^ T cells stratified for CD20 or CD103 positivity did not differ between MS lesional and normal-appearing WM (Figures S12 and S13).

More recently, CD4^+^ T cells expressing CCR5, CCR6, CXCR5, or granzyme K and sharing characteristics of T-helper type 1/17 (T_H_1/17) cells have been shown to home specifically into the CNS of MS patients.^21^^,^^22^^,^^23^^,^^24^ We compared proportions of cells positive for CCR2, CCR5, CCR6, CXCR5, granzyme K, and CD25 between non-MS WM and MS normal-appearing and lesional WM CD8^+^ and CD4^+^ CD69^+^ T cells (Figures 6B and S4). In accordance with the gene expression data, we did not find a specific MS lesional enrichment of T cells positive for these markers.

To further explore potential functional differences, we stimulated lymphocytes derived from non-MS WM and MS normal-appearing and lesional WM *ex vivo* with phorbol 12-myristate 13-acetate/ionomycin. Notably, intracellular staining of cytokines IL-2, TNF, and IFNγ showed decreased expression in MS lesional compared to normal-appearing MW CD8^+^ and CD4^+^ CD69^+^ T cells, with an increased proportion of cells producing neither of these cytokines (Figure 6C). In line with the reported polyfunctionality of brain T cells,^8^ cytokine-producing T cells were often triple-positive (IL-2^+^ TNF^+^ IFNγ^+^). The reduction of cytokine production did not coincide with a lower cell viability, as measured with Apotracker Green (Figure S14).

We hypothesized that this lack of cytokine production could be due to exhaustion of T cells residing in the local, presumably chronic inflammatory environment of mixed active/inactive MS lesions. However, in line with the gene expression data, protein expression of (combinations of) inhibitory receptors associated with T cell exhaustion did not differ between MS lesional and normal-appearing WM CD8^+^ and CD4^+^ CD69^+^ T cells^25^ (Figure 6D and S4, and data not shown). Accordingly, because CD8^+^ T cell exhaustion is also associated with enhanced granzyme B production,^26^ we previously found no increase in granzyme B expression by MS lesional compared to normal-appearing MW CD8^+^ T cells.^17^

### Increased OPN expression in MS lesions corresponds with decreased cytokine production by T cells

Next, we hypothesized differences in tissue organization to determine cytokine production by MS lesional versus normal-appearing WM T cells. Because we here studied T cells obtained from active and mixed active/inactive MS lesions, these cells likely contained larger proportions originating from the parenchyma rather than the PVS.^17^ Applying immunohistochemistry for CD3, TNF, and GFAP (glial fibrillary acidic protein) to show the PVS border, we repeated this finding, as the proportion of T cells located in the parenchyma was significantly higher in MS active and mixed active/inactive lesional compared to inactive lesional and normal-appearing WM (Figures 6E and 6F). Further, we noted a lower percentage of TNF^+^ T cells in the parenchyma compared to the PVS in MS donors (Figure 6G). This finding supports earlier finding that the brain parenchyma provide anti-inflammatory signals,^27^ which can suppress T cell cytokine production.

**Figure 7:**
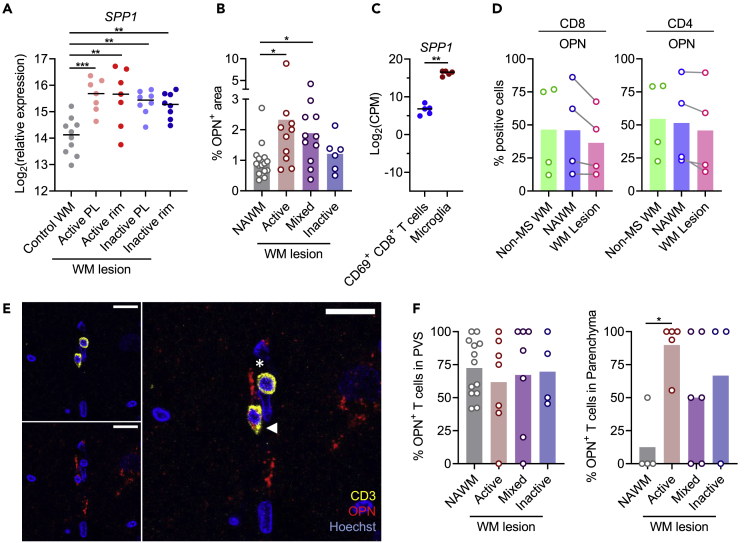
OPN expression in MS lesions (A) *SPP1* gene expression in MS lesional compared to control WM. Data extracted from Hendrickx et al.^29^ One way ANOVA was used with a post-hoc Tukey’s test, and p values are shown in the plots. PL, perilesional. (B) OPN expression in MS active and mixed active/inactive lesional compared to normal-appearing WM, determined by immunofluorescence staining. Pairwise comparisons using estimated marginal means were used, and p values are shown in the plots. (C) Gene expression of *SPP1* in microglia and CD8^+^ CD69^+^ T cells from subcortical WM, determined by RNA sequencing in n = 5 brain donors (second dataset). CPM, counts per million. Mann-Whitney U test was used, and p value is shown in the plots. (D) Percentage of OPN-expressing brain CD8^+^ and CD4^+^ T cells in MS normal-appearing and lesional WM, determined by flow cytometry. (E) Representative immunofluorescence staining image of T cells in contact with (asterisk) or expressing (arrowhead) OPN. (F) Percentage of OPN^+^ T cells in the PVS (left panel) and parenchyma (right panel) of MS normal-appearing and lesional WM, determined by immunofluorescence staining. Pairwise comparisons using estimated marginal means were used, and p values are shown in the plots. ∗p<0.05; ∗∗p<0.01; ∗∗∗p<0.001.

The parenchyma provides an environment enriched for inhibitory signals, compared to the PVS.^27^ Accordingly, OPN has been reported to act as an immune checkpoint to suppress T cell activation.^28^*SPP1* gene expression is upregulated in MS lesional tissue^29^ (Figure 7A), and various recent studies attributed this upregulation to microglia.^30^^,^^31^^,^^32^ We validated this in our donors with immunohistochemistry on protein level, as within sections, the OPN-positive area was higher in MS lesional compared to normal-appearing WM (Figures 7B and S15). RNA sequencing of n = 5 corresponding samples revealed higher gene expression of *SPP1* in microglia compared to brain T cells (Figure 7C). The latter did not upregulate OPN production in MS lesions (Figures 7D and S11). By immunofluorescence staining of MS tissue, we again observed a high (68 ± 28%) percentage of T cells in contact with OPN, both in MS lesional as well as in normal-appearing WM T cells (Figure 7E). Focusing on parenchymal T cells, MS lesional T cells were more often associated with OPN compared to T cells in the normal-appearing WM (Figure 7F).

## Discussion

Here, we provide evidence that human brain CD8^+^ and CD4^+^ T cells display a T_RM_-cell transcriptional profile characterized by the expression of *SPP1* (OPN) and *MS4A1* (CD20). Perivascular brain T_RM_ cells showed a spatial association with OPN depositions, possibly controlling compartmentalization and activation of these cells. In MS lesional WM, brain CD8^+^ and CD4^+^ T cells retained their T_RM_-cell transcriptomic profile, showing few DE genes compared to normal-appearing WM and no common traits across groups. MS lesional expression and deposition of OPN was upregulated and associated with a reduced pro-inflammatory cytokine production by MS lesional brain T_RM_ cells. This lowered cytokine production did not associate with an increased expression of inhibitory receptors and/or markers of exhaustion in MS lesional versus normal-appearing WM brain T_RM_ cells. Altogether, our data indicate that brain T cells physiologically reside in a highly controlled environment, which limits their continuous contribution to pathological, focal inflammation within chronic MS lesions in the brain parenchyma. These setting may dictate approaches to control the contribution of these cells to early lesion development or expansion in progressive MS. Disease-modifying treatments thus far are of limited effectiveness in suppressing disability progression without relapses in progressive MS,^33^ and remyelination strategies have not been successful in clinical trials yet.^34^ An explanation could be the inability of these treatments to either reach or control focal, compartmentalized inflammatory processes in progressive MS. Local expression of OPN in addition to other regulatory molecules,^27^ may add to the control of brain-resident lymphocyte activation in the PVS and parenchyma of people with MS.

Previously, we showed with multi-color flow cytometry of viable brain lymphocyte fractions acquired with rapid postmortem autopsies that human WM is populated by CD8^+^ and CD4^+^ memory T cells with a T_RM_-cell phenotypic profile.^8^^,^^9^^,^^17^ In our current work, we expand on these data, showing with bulk RNA sequencing that the brain WM and GM CD8^+^ and CD4^+^ T cell transcriptome harbors a core T_RM_-cell expression profile. Although this technique does not allow an analysis of phenotypical diversity of this brain T cell pool, it allows an in-depth characterization of the transcriptional features of brain-homing T cells. The gene set enrichment analysis supports a downregulation of non-T_RM_-cell associated gene sets with also an upregulation of T_RM_-cell associated genes. The direct comparison of the brain T_RM_ cells with circulating T_EM(RA)_ cells is likely influenced by unique characteristics of brain-resident T cells. Their phenotype shows similarities with T cells isolated from other tissues, such as lungs, skin, gut, and liver.^10^^,^^13^^,^^14^ The notable *SPP1* gene activity seen by us in brain T cells is not a general trait of T_RM_ cells^13^^,^^35^ but has been found before in T cells isolated from salivary glands,^36^ which may indicate that OPN production could be acquired by T cells in other tissues as well. Alternatively, high *SPP1* expression has been identified as characteristic of senescence-associated T cells, which could point toward a chronically activated state of brain T cells.^37^ The expression of *MS4A1* (CD20) by brain T cells could be a more unique feature of brain-homing T cells or cells associated with inflammatory diseases. Since the first detailed description of this cell type in MS by Palanichamy et al., CD20^dim^ T cells attracted much attention.^38^ Our current findings corroborate our earlier work, showing an expanded proportion of CD20^dim^ T cells among the brain CD8^+^ T cell fraction. We showed that brain CD20^dim^ CD8^+^ T cells more abundantly express the chemokine receptor CXCR6, the activation marker Ki-67, and the lytic mediator granzyme B, compared to their CD20^neg^ counterparts.^15^ Recent findings show that CD8^+^ memory T cell populations in MS CSF are characterized by high expression of CD69, granzyme K, and CD20, and could represent brain T_RM_-cell precursors.^39^ Furthermore, von Essen et al. showed previously that the proportion of CD20^dim^ CD4^+^ and CD8^+^ T cells is enriched in CSF, compared to circulating T cells, that CD20^dim^ T cell fractions can be found across differentiation stages (i.e. naive and memory cells), and that CD20^dim^ T cells outside the CNS are also enriched in the expression of integrins and chemokine receptors (CD49d, CCR2, CCR5, CCR6, CXCR3), produce more abundantly pro-inflammatory cytokines (IFNγ, TNF, GM-CSF), and possess a higher responsiveness to myelin antigens (MOG/MBP).^40^ We show that several of these phenotypic characteristics are retained by brain CD20^dim^ CD8^+^ and CD4^+^ T_RM_ cells. The presence of CD20, as we currently show and previously showed with RT-qPCR,^15^ makes trogocytosis of CD20 on B–T cell interaction less likely as exclusive explanation for the existence of this cell population.^41^ The observation that MS therapies as rituximab and alemtuzumab deplete circulating CD20^dim^ T cells^38^^,^^40^ suggests that these treatments may also affect long-term development and maintenance of these cells in the PVS. Accordingly, selective depletion of CD20^dim^ T cells also ameliorates experimental models of neuroinflammation.^41^ Because we previously found CD20^dim^ T cells enriched in MS tissue compared to control tissue,^15^ depletion of these cells could potentially explain effects of CD20-directed therapies on long-term disease progression.

Our current data show that increased *SPP1* (OPN) expression is a generic key characteristic of brain T_RM_ cells, in contrast to circulating T_EM(RA)_-cell fractions. Several CNS-resident cell populations have been shown to express OPN, such as microglia, neuronal cell bodies, and oligodendrocytes. In addition, OPN has previously been identified as a trait of CNS-homing lymphocytes and hematologic malignancies.^42^ This abundant expression of OPN across cell types suggests it as a constitutive element of cellular homeostasis and interaction within the CNS. OPN can be found in both an intracellular and a secreted form, the latter being processed by thrombin cleavage among other post-translational modifications.^43^ These different conformations of OPN mediate distinct biological functions as well as possibility to engage in molecular interaction with, among others, the integrin CD49d (VLA-4) and the scavenger receptor CD44. In this sense, both pro-and anti-inflammatory characteristics have been attributed to OPN, including a more pro-inflammatory role in T cell homeostasis and a more anti-inflammatory role in myeloid cell activation.^44^ However, recent work by Klement et al. suggests that OPN may also serve as a checkpoint inhibitor controlling local CD8^+^ T cell (re)activation.^28^

We think our data revisits the role of OPN in MS. Previously, soluble OPN levels have been shown to be upregulated in blood of people with relapsing MS^45^^,^^46^^,^^47^ and in CSF of people with progressive MS. Accordingly, Chabas et al. showed an upregulation of *SPP1* transcription in association with MS lesions, and staining for OPN of microvascular endothelial cells, macrophages, astrocytes, and microglia in MS tissue.^48^ Sinclair et al. showed that this increased expression co-localizes with demyelination and microglial activation.^49^ Acknowledging the role of OPN as a pro-inflammatory T cell cytokine, Chabas et al. showed in *Spp1*-knockout mice a reduction of experimental autoimmune encephalomyelitis (EAE) severity and chronicity.^48^ However, the EAE model depends on a profound recruitment of auto-reactive T cells from secondary lymphoid organs, which is likely much more dependent on immune regulatory processes outside the CNS, compared to the local inflammatory process of advanced MS. In *Spp1*-knockout mice, circulating T cells showed reduced proliferation and IFNγ production together with an increase in IL-10 expression, ultimately culminating in reduced severity of disease within the CNS.^48^ The data of Klement et al. warrant a reappraisal of OPN in advanced MS.^28^ We previously showed that brain CD8^+^ T_RM_ cells in MS lesions upregulate expression of CD44,^17^ and now we demonstrate that MS lesional T cells retain expression of OPN, that brain T cells spatially distribute in close contact with these OPN depositions, and that OPN expression and deposition are increased in MS lesions. The source of these depositions could be T cells, but also microglia, which we show here to contain much higher levels of *SPP1* transcripts. MS-associated microglia further upregulate *SPP1* gene activity,^30^^,^^31^^,^^32^ and OPN has been identified as a chemoattractant for both myeloid and T cell populations.^44^ Whether these OPN depositions contribute to a more anti-inflammatory milieu in chronic MS lesions, culminating directly or indirectly in a reduced cytokine response of T cells, remains to be further studied. Therefore, increased local OPN expression in advanced MS could be regarded as a compensatory anti-inflammatory process, rather than a detrimental pro-inflammatory T cell cytokine response. This notion could offer new approaches to study the role of OPN in the local control of inflammation. In addition to this, enhancement of remyelination and neuroregeneration by OPN has also been suggested in experimental models of demyelination.^50^

Because we observed few DE genes in MS lesional versus normal-appearing WM brain T_RM_ cells, our previously hypothesized relevance of these cells for progressive MS can be disputed.^51^ Accordingly, disease-modifying therapies affecting peripheral T cell homeostasis have a limited effect on progressive MS, and T cell-associated risk genes are primarily associated with the risk of developing MS,^20^ while their association with MS progression is currently under investigation. However, there are several alternative hypotheses to consider. First, we studied T cells in chronic mixed active/inactive lesions, while these cells may rather contribute to MS lesion formation or expansion in surrounding normal-appearing WM. We previously showed changes in microglial gene expression in normal-appearing WM surrounding MS lesions,^29^ suggesting cellular changes are already taking place. Brain T_RM_ cells from these regions were not studied in our current project. Second, discrete sub-populations of brain T_RM_ cells could contribute to MS lesion formation, which remain hidden in the total brain T cell transcriptome obtained by our bulk RNA sequencing approach. Previously, Van Langelaar et al. showed that circulating CD4^+^ T cells with a high expression of CCR6 (Th17.1 cells) have a higher propensity to be recruited to the CNS of people with MS and are preferentially retained in the circulation by natalizumab treatment.^21^ Recently, Kaufman et al. showed that natalizumab-treatment results in a CD161^+^ CD4^+^ T cell population with several similar characteristics being retained in the circulation, and that low-frequent hits of cells with a similar transcriptome could be revealed with spatial RNA sequencing among local T cell infiltrates in chronic MS.^23^ Accordingly, Herich et al. also showed that granzyme K^+^ CCR5^+^ CD4^+^ T cell fractions can be isolated from resected WM samples of donors being treated with epilepsy surgery, and that these cells are clonally expanded in the circulation of people with MS.^22^ Regarding CD8^+^ T cells, Beltrán et al. showed that in CSF of twins discordant for MS, CD8^+^ T cell fractions with a T_RM_-cell like profile were clonally expanded in the CSF of the twin without MS.^18^ Such small sub-populations may have remained under the radar in our current study. However, it also remains to be seen whether a phenotypic or transcriptomic profile of circulating T cell clones is preserved in T cells entering and residing in tissue. A paired analysis of TCR clones and phenotypes in several compartments (i.e., circulation, CSF, tissue) could bridge these gaps. Third, we may encounter cells that have contributed to lesion progression in MS, but are in end-stage MS in an exhausted state. Exhausted CD8^+^ T cells are characterized by impaired cytokine production (most notably IL-2), retained lytic enzyme expression, and co-expression of by itself nonspecific inhibitory receptors.^52^ We could not find an increased co-expression of inhibitory receptors in the brain T_RM_-cell transcriptome. And although we could demonstrate with flow cytometry sub-populations of PD-1^+^CD39^+^ T cells within MS normal-appearing and control WM, these were not enriched in MS lesions, did not express markers as CTLA-4, TIM-3, and LAG-3, and did not account for the increased proportion of cells not producing cytokines in MS lesions. Therefore, these cells do not display at least a fully exhausted phenotype. Fourth, Machado-Santos et al. hypothesized that T cells may contribute to lesion formation and progression in MS rather by making pro-inflammatory mediators from the PVS.^16^ We previously showed that a substantial proportion of MS lesional T cells leaves the PVS and enters the brain parenchyma in MS, where inhibitory receptors on these cells may encounter their ligands, such as PD-L1^8^ and, as discussed above, possibly also OPN. Our data now show that lesional WM T cells exhibit functional enrichments related to cell damage and repair, whereas normal-appearing WM-associated T cells associate with more pro-inflammatory gene sets, such as cell–cell contact and response to antigenic stimulation. This observation provides further support for the PVS as immunogenic compartment compared to the highly controlled environment of the brain parenchyma.^2^ Taken together, a mix of activated perivascular and controlled parenchymal T cells could dilute DE genes within the bulk transcriptome.

In sum, we here provide a detailed characterization of the phenotypic, transcriptional, and functional profile of brain CD8^+^ and CD4^+^ T cells, which will help to understand their role in physiological immune surveillance of the CNS, but also in pathological conditions. Our data suggest an important role of OPN in the control and compartmentalization of brain T cells, which should be investigated further for the benefit of people with advanced MS.

### Limitations of the study

Limitations of the current study are first the analysis of postmortem tissue of elderly human brain donors with advanced MS. The direct applicability of our data on donors earlier in their disease course remains not entirely certain,^17^ yet Beltrán et al. described CD8^+^ T_RM_-like cells in the CSF during possibly the earliest stages of MS in MS discordant twins.^18^ In addition, our data provide most information on the disease process in advanced progressive MS of which treatment is an unmet clinical need. Second, we show associations between disease status and brain T cell characteristics, yet do not provide functional or mechanistic studies. Also, direct comparisons cannot be made between the MS and control datasets because biological differences are confounded with possible technical differences between the two batches. Third, we studied the bulk transcriptome of brain T cells, in which relevant (clonally expanded) T cell subsets are likely hidden,^53^^,^^54^ which could be revealed with techniques as single-cell or single-nuclear RNA sequencing. Nevertheless, bulk RNA sequencing provides the most deep and profound characterization of the T cell transcriptome, and potential sub-populations were in the current study addressed with flow cytometry. The strengths of this study are the unique analysis of viable T cells from human brain tissue of a large sample of postmortem brain donors, the deep transcriptional profiling of these cells, and the combination of different techniques as RNA sequencing, flow cytometry, immunohistochemistry, and immunofluorescence to study the characteristics of brain T cells.

## STAR★Methods

### Key resources table

**Table:** 

REAGENT or RESOURCE	SOURCE	IDENTIFIER
**Antibodies**		

3,30-diaminobenzidine (DAB)	DAKO	Cat#K5007
Anti-human CD3	DAKO	Cat#A0452 RRID: AB_2335677
Anti-human CD3–PEcy5.5	eBioscience	Cat#35-0036-42 RRID: AB_11220085
Anti-human CD3–PE-CF594	eBioscience	Cat#14-0038-82 RRID: AB_467059
Anti-human CD4–Alexa Fluor 700	BD Biosciences	Cat#557922 RRID: AB_396943
Anti-human CD4–BUV563	BD Biosciences	Cat#612912 RRID: AB_2739451
Anti-human CD8–BV786	BD Biosciences	Cat#563823 RRID: AB_2687487
Anti-human CD8–BV421	BioLegend	Cat#301036 RRID: AB_10960142
Anti-human CD20–APC	BioLegend	Cat#302310 RRID: AB_314257
Anti-human CD25–BV421	BioLegend	Cat#302630 RRID: AB_11126749
Anti-human CD28	In-house	Clone 15E8
Anti-human CD29	In-house	Clone TS2/16
Anti-human CD39–FITC	BioLegend	Cat#328206 RRID: AB_940423
Anti-human CD45RA–BV650	BioLegend	Cat#304136 RRID: AB_2563653
Anti-human CD69–BV650	BioLegend	Cat#310934 RRID: AB_2563158
Anti-human CD103–BUV395	BD Biosciences	Cat#564346 RRID: AB_2738759
Anti-human CD103–PE	eBioscience	Cat#12-1038-42 RRID: AB_11150242
Anti-human CD159a/NKG2A–PEcy7	BioLegend	Cat#375114 RRID: AB_2888865
Anti-human CD185/CXCR5–Alexa Fluor 488	BD Biosciences	Cat#558112 RRID: AB_397034
Anti-human CD192/CCR2–BV510	BioLegend	Cat#357218 RRID: AB_2566504
Anti-human CD195/CCR5–Alexa Fluor 700	BioLegend	Cat#313713 RRID: AB_528761
Anti-human CD196/CCR6–PEcy7	BioLegend	Cat#353418 RRID: AB_10916518
Anti-human CD197/CCR7–BUV395	BD Biosciences	Cat#563977 RRID: AB_2738519
Anti-human CD197/CCR7–PEcy7	BD Biosciences	Cat#557648 RRID: AB_396765
Anti-human CD223/LAG3–BV510	BioLegend	Cat#369318 RRID: AB_2715781
Anti-human CD244/2B4–Alexa Fluor 700	BioLegend	Cat#329526 RRID: AB_2687332
Anti-human CD279/PD-1–PerCP-eFluor710	BD Biosciences	Cat#46-2799-42 RRID: AB_1834415
Anti-human CD366/TIM3–BV421	BioLegend	Cat#345008 RRID: AB_11218598
Anti-human GFAP	Santa Cruz Biotechnology	Cat#sc-6170 RRID: AB_641021
Anti-human granzyme K–PerCP-eFluor710	eBioscience	Cat#46-8897-42 RRID: AB_2573854
Anti-human IFNγ–FITC	BD Biosciences	Cat#340449 RRID: AB_400425
Anti-human IL-2–PEcy7	BD Biosciences	Cat#560707 RRID: AB_1727542
Anti-human osteopontin (OPN)	Santa Cruz Biotechnology	Cat#sc-21742 RRID: AB_2194997
Anti-human osteopontin (OPN)–PE	R&D Systems	Cat#IC14331P RRID: AB_10891538
Anti-human TNF–unconjugated	Proteintech	Cat#60291-1-Ig; RRID: AB_2833255
Anti-human TNF–Alexa Fluor 700	BD Biosciences	Cat#557996 RRID: AB_396978
Apotracker Green	BioLegend	Cat#427402
Donkey-anti-goat–Alexa Fluor 488	Jackson ImmunoResearch	Cat#705-545-003 RRID: AB_2340428
Donkey-anti-rabbit–Cy3	Jackson ImmunoResearch	Cat#711-165-152 RRID: AB_2307443
LIVE/DEAD Fixable Viability Dye eFluor 780	Thermo Fisher Scientific	Cat#65-0865
FcR Blocking Reagent	Miltenyi Biotec	Cat#130-059-901 RRID: AB_2892112
Hoechst 33342	Thermo Fisher Scientific	Cat#H3570
Horse-anti-mouse–biotin	Vector Laboratories	Cat#BA-2000 RRID: AB_2313581
Streptavidin–Cy5	Jackson ImmunoResearch	Cat#016-170-084 RRID: AB_2337245
Tyramide–biotin	Sigma-Aldrich	Cat#SML2135
VECTASTAIN Elite ABC HRP Kit	Vector Laboratories	Cat#PK-6100 RRID: AB_2336819

**Biological samples**		

Human brain tissue and blood	Netherlands Brain Bank	https://www.brainbank.nl/

**Chemicals, peptides, and recombinant proteins**		

Cytofix/Cytoperm Fixation and Permeabilization Solution kit	BDBiosciences	Cat#554722
Brefeldin A	Sigma-Aldrich	Cat#B7651
Collagenase I	Worthington Biochemical Corporation	Cat#LS004197
Collagenase IV	Worthington Biochemical Corporation	Cat#LS004189
DMEM, high glucose, HEPES, no phenol red	Thermo Fisher Scientific	Cat#21063
DNAse I	Roche	Cat#11284932001
Hibernate A	Thermo Fisher Scientific	Cat#A1247501
Ionomycin	Sigma-Aldrich	Cat#I0634
Lymphoprep	STEMCELL Technologies	Cat#1114544
Penicillin-streptomycin	Thermo Fisher Scientific	Cat#15140
Percoll	GE Healthcare	Cat#GE17-0891-01
Phorbol 12-myristate 13-acetate (PMA)	Sigma-Aldrich	Cat#P8139
Protein transport inhibitor (Monensin)	BD Biosciences	Cat#554724
UltraComp eBeads Plus Compensation Beads	Thermo Fisher Scientific	Cat#01-3333-42

**Critical commercial assays**		

RNAeasy micro kit	Qiagen	Cat#74004

**Deposited data**		

Bulk RNA sequencing data	This paper	GEO: GSE216030

**Software and algorithms**		

AutoAnnotate (version 1.3.5, Cytoscape plugin)	Kucera et al.^55^	https://apps.cytoscape.org/apps/autoannotate
Bioconductor (version 3.15)	Huber et al.^56^	https://bioconductor.org/
biomaRt (version 2.51.4, R package)	Durinck et al.^57^	Bioconductor
Cytoscape (version 3.9.0)	Shannon et al.^58^	https://cytoscape.org/
edgeR (version 3.37.4, R package)	Robinson and Oshlack^59^	Bioconductor
emmeans (version 1.8.0, R package)	N/A	https://CRAN.R-project.org/package=emmeans
EnrichmentMap (version 3.3.3, Cytoscape plugin)	Merico et al.^60^	https://apps.cytoscape.org/apps/enrichmentmap
fastp (version 0.23.2)	Chen et al.^61^	https://github.com/OpenGene/fastp
FastQC (version 0.11.9)	Babraham Institute	https://www.bioinformatics.babraham.ac.uk/projects/fastqc/
FlowJo (version 10.8.1)	BD Biosciences	https://www.flowjo.com/
ggplot2 (version 3.3.5, R package)	Wickham^62^	https://CRAN.R-project.org/package=ggplot2
HISAT2 (version 2.2.1)	Kim et al.^63^	http://daehwankimlab.github.io/hisat2/
HTSeq (first and third datasets: version 1.99.2; second dataset: version 2.0.2)	Anders et al.^64^	https://htseq.readthedocs.io/en/master/
limma (version 3.51.8, R package)	Law et al.^65^	Bioconductor
lme4 (version 1.1-29, R package)	N/A	https://github.com/lme4/lme4/
PCAtools (version 2.8.0, R package)	https://github.com/kevinblighe/PCAtools	Bioconductor
Prism (version 9.4.1)	GraphPad Software	N/A
QuPath (version 0.2.3)	Banhead et al.^66^	https://qupath.github.io/
Trimmomatic (version 0.39)	Bolger et al.^67^	http://www.usadellab.org/cms/?page=trimmomatic
R (version 4.2.0)	The R Project	https://www.r-project.org/

**Other**		

Ensembl (version 106)	EMBL-EBI	https://www.ensembl.org/index.html
Molecular Signatures Database (MSigDB, version 7.0)	Subramanian et al.^68^	https://www.gsea-msigdb.org/gsea/msigdb/index.jsp
NovaSeq6000 platform	Illumina at GenomeScan B.V.	N/A
Quickomics Resource for the first dataset	Bxgenomics	http://quickomics.bxgenomics.com/?serverfile=Quickomics_BrainTcell_Circulating
Quickomics Resource for the second dataset	Bxgenomics	http://quickomics.bxgenomics.com/?serverfile=Quickomics_BrainTcell_Location
Quickomics Resource for the third dataset	Bxgenomics	http://quickomics.bxgenomics.com/?serverfile=Quickomics_BrainTcell_MS

### Resource availability

#### Lead contact

Further information and requests for resources and reagents should be directed to and will be fulfilled by the lead contact, Jörg Hamann (j.hamann@amsterdamumc.nl).

#### Materials availability

This study did not generate new unique reagents.

### Experimental model and subject details

#### Post-mortem brain tissue and blood

Human brain tissues and blood were provided by the Netherlands Brain Bank (Amsterdam, The Netherlands; https://www.brainbank.nl). Informed consent for performing autopsy and using tissue and clinical data for research purposes was obtained from brain donors and approved by the Ethics Committee of Amsterdam UMC (Location VUmc, Amsterdam, The Netherlands).

White matter (WM) and gray matter (GM) tissue blocks were collected from corpus callosum (n = 11) and occipital cortex (n = 11) (first dataset) as well as from subcortex (n = 5), corpus callosum (n = 3), and medulla (n = 4) (second dataset), respectively. Normal-appearing WM (n = 6), normal-appearing GM (n = 6), lesional WM (n = 5), and lesional GM (n = 2) tissue blocks of MS donors were dissected at autopsy on post-mortem magnetic resonance imaging guidance^69^ (third dataset).

Neurological diagnosis was confirmed post-mortem by a neuropathologist, based on both clinical and pathological data. Non-neurological control donors with cognitive problems, based on clinical data, were excluded from the analysis. Brain donor characteristics are displayed below.

**Table:** Brain donors and sample information for RNA sequencing

Brain donor	Diagnosis	Tissue	Sex	Age (years)	PMD (h:min)	pH CSF	Brain weight (g)	Cause of death
1	MS	CC WM, OCC GM, blood	F	63	6:05	6.5	1130	Ceased oral intake
2	MDD	CC WM, OCC GM, blood	F	72	8:45	6.5	1265	Legal euthanasia
3	HC	CC WM, OCC GM, blood	F	102	4:55	6.7	1100	Old age
4	MSA	CC WM, OCC GM, blood	M	55	8:00	6.6	1535	Multiple system atrophy
5	HC	CC WM, OCC GM, blood	F	98	8:45	6.3	1010	Heart failure
6	PD	CC WM, OCC GM, blood	M	61	7:00	6.6	1,485	Possible aspiration
7	PD	CC WM, OCC GM, blood	M	65	4:50	7.6	1350	Respiratory insufficiency
8	FTD	CC WM, OCC GM, blood	M	49	5:05	6.3	1105	Dehydration and cachexia
9	AD	CC WM, OCC GM, blood	F	94	5:35	6.2	930	General deterioration, dehydration, and ceased oral intake
10	HC	CC WM, OCC GM, blood	F	79	6:00	6.8	1075	Lung cancer
11	AD	CC WM, OCC GM, blood	F	94	5:10	6.4	1155	Lung cancer
12	FTD	Sub WM, CC WM, Medulla WM and GM	M	66	5:50	6.5	1385	Myocardial infarction
13	Narco	Sub WM, CC WM, Medulla WM and GM	M	75	5:49	6.6	1246	Euthanasia
14	FTD	Sub WM, Medulla WM and GM	M	69	4:55	6.6	1306	Myocardial infarction
15	AD	Sub WM, Medulla WM and GM	M	75	5:40	6.6	1211	Ceased oral intake, palliative sedation
16	FTD	Sub WM, CC WM, Medulla WM and GM	M	73	6:40	6.0	1269	Cardiac arrest
17	MS	NAWM, NAGM, lesional WM	M	73	5:40	6.3	1270	Legal euthanasia
18	MS	NAWM, NAGM, lesional WM	F	66	5:40	6.5	1160	N/A
19	MS	NAWM, lesional WM	F	61	6:25	6.2	1055	Wound infection and pneumonia
20	MS	NAWM, NAGM, lesional WM	M	85	8:45	6.9	1235	Legal euthanasia
21	MS	NAWM, NAGM, lesional WM, lesional GM	F	71	6:20	6.7	1335	Legal euthanasia
22	MS	NAWM, NAGM, lesional WM, lesional GM	F	66	8:00	6.3	1305	Pneumonia

Samples 1 to 11, 12 to 16, and 17 to 22 refer to the three datasets of this study. AD: Alzheimer’s disease, Age: age at death, CC: corpus callosum, CSF: cerebrospinal fluid, F: female, FTD: frontotemporal dementia, GM: gray matter; HC: healthy control, M: male, MDD: major depressive disorder, MS: multiple sclerosis, MSA: multiple system atrophy, n/a: data not available, NAGM: normal-appearing (perilesional) GM, Narco: narcolepsy, NAWM: normal-appearing (perilesional) WM, OCC: occipital cortex, PD: Parkinson’s disease, PMD: post-mortem delay, Sub: subcortical, WM: white matter.

Fresh WM tissue of n = 10 control and n = 9 MS donors from the Netherlands Brain Bank was used for flow cytometry.

**Table:** Brain donor and sample information for flow cytometry

Brain donor	Diagnosis	Tissue	Sex	Age (years)	PMD (h:min)	pH CSF	Brain weight (g)	Cause of death
23	MS	NAWM, NAGM, Lesional WM, Lesional GM, blood	F	73	7:45	6.5	1,080	Legal euthanasia
24	HC	WM, GM, blood	F	99	4:05	6.7	1130	Legal euthanasia
25	PD	WM, GM, blood	M	84	5:30	N/A	1354	N/A
26	MS	NAWM, NAGM, lesional WM, blood	M	71	6:55	6.4	1310	Respiratory insufficiency
27	HC	WM, GM, blood	F	88	4:30	6.7	924	Legal euthanasia
28	PSP	WM, GM, blood	F	84	4:50	6.8	1120	Ceased oral intake
29	MS	NAWM, NAGM, Lesional WM, Lesional GM, blood	F	60	5:05	6.8	1192	Legal euthanasia
30	MS	NAWM, lesional WM, blood	M	51	6:05	6.55	1085	Dehydration, general deterioration in MS
31	LBV	WM, GM, blood	M	71	4:15	6.6	1400	Legal euthanasia
32	PD	WM, GM, blood	F	79	3:45	6.7	1027	Legal euthanasia
33	PD	WM, GM, blood	F	77	11:05	6.8	1285	Legal euthanasia
34	PTSS	WM, GM, blood	F	60	3:48	6.8	1160	Legal euthanasia
35	MS	NAWM, NAGM, lesional WM, blood	F	65	9:30	6.7	1275	Sepsis
36	MSA	WM, GM, blood	F	69	5:20	6.6	1135	Legal euthanasia
37	HC	WM, GM, blood	F	85	7:30	6.5	1005	Ceased oral intake, pleural fluid, liver metastases (unknown primary tumor)
38	MS	NAWM, NAGM, esional WM, blood	F	71	6:50	6.4	1310	Pneumonia
39	MS	NAWM, NAGM, lesional WM, blood	F	59	7:45	N/A	N/A	Dehydration and swallowing problems due to MS
40	MS	NAWM, lesional WM, blood	M	57	4:45	6.6	1390	Euthanasia
41	MS	NAWM, lesional WM, blood	F	54	5:30	N/A	1325	Euthanasia

Samples 1 to 17 were used for protein expression and functional profiling, samples 18 and 19 were used for detecting apoptotic cells. Age: age at death, CSF: cerebrospinal fluid, F: female, GM: gray matter, HC: non-neurological healthy control, LBV: Lewy body variant of Alzheimer’s disease, M: male, MS: multiple sclerosis, MSA: multiple system atrophy, n/a: data not available, NAGM: normal-appearing (perilesional) GM, NAWM: normal-appearing (perilesional) WM, PD: Parkinson’s disease, PMD: post-mortem delay, PSP: progressive supranuclear palsy, PTSS: post-traumatic stress disorder, WM: white matter.

Formalin-fixed, paraffin-embedded WM tissue sections of n=10 control and n=17 MS donors from the Netherlands Brain Bank from were used for immunohistochemistry.

**Table:** Brain donor and sample information for immunohistochemistry

Diagnosis	Cases (n)	Age (years)	Sex	PMD (h:min)	pH CSF	Brain weight (g)
MS	17	60.7 ± 9.7	6F/11M	8:58 ± 1:27	6.6 ± 0.3	1229 ± 112
Non-neurological controls	10	79.1 ± 8.2	4F/6M	6:53 ± 2:31	6.6 ± 0.2	1257 ± 163

F: female, M: male, MS: multiple sclerosis, PMD: post-mortem delay.

### Method details

#### Isolation of mononuclear cells from brain and peripheral blood

Brain tissue blocks of 4–6 g were dissected at autopsy and stored in a 50-mL tube containing 25 mL Hibernate A medium at 4°C until further processing. Mononuclear cells were isolated as described before.^70^ Brain tissue was put in a 10-cm petri dish with Hibernate A medium, and large vessels and membranes were removed. WM tissue was chopped into little chunks using two scalpels (Sigma-Aldrich). GM tissue was grinded over a 100-mesh tissue sieve (Sigma-Aldrich) with a plunger from a 50-mL disposable syringe. Tissue chunks were pipetted into a 50-mL tube, followed by enzymatic digestion with 300 U/mL collagenase I (for RNA sequencing samples) or 300 U/mL collagenase IV (for flow-cytometric samples) in Hibernate A medium supplemented with 33 μg/mL DNAse I for 60 min in a water bath at 37°C. Every 15 min, the tubes were vortexed. After incubation, the cell suspensions were filtered through a clean 100-mesh tissue sieve into a 10-cm petri dish, collected in 50-mL tubes, and filled up to 50 mL with cold DMEM medium (450 mL DMEM without phenol, 50 mL fetal bovine serum, and 5 mL penicillin G and streptomycin mix). The tubes were centrifuged at 1,800 rpm for 10 min at 4°C, after which the supernatant was discarded and the pellets were resuspended in ∼15 mL cold DMEM medium, resulting in cell suspensions in a final volume of 20 mL. 10 mL cold Percoll was slowly dripped directly on the cell suspensions after which the tubes were centrifuged at 4,000 rpm for 30 min at 4°C with the acceleration set at 5 and the brake set at 4. Three layers were obtained with myelin at the top, erythrocytes at the bottom, and mononuclear cells in between. Mononuclear cells were collected from the interlayer into a 50-mL tube using a glass pipette (after the myelin had either been removed with a pipette or pushed carefully aside). Cells were washed by filling up the tube with cold DMEM medium followed by centrifugation at 1,500 rpm for 10 min at 4°C. In parallel with collecting the brain tissue, 5 mL peripheral blood was collected by cardiac puncture from the deceased donors and stored in a Vacutainer EDTA tube (BD) at room temperature. Mononuclear cells were isolated using Lymphoprep according to the manufacturers’ instructions. Erythrocytes in cell suspensions of mononuclear cells from brain and peripheral blood were removed by erythrocyte lysis buffer, and cells were wash twice by cold DMEM medium.

#### RNA isolation and sequencing

Blood CD8^+^ and CD4^+^ CCR7^−^CD45RA^−^ effector memory T (T_EM_) and CCR7^−^CD45RA^+^ effector memory re-expressing CD45RA T (T_EMRA_) cells and brain CD8^+^ and CD4^+^ CD69^+^ T cells were sorted on a BD FACSAria II cell sorter (BD Biosciences) (for staining procedure see below). Gating strategies are shown in Figure S1. Sorted cells were lysed in 350 μL buffer RLT plus 1% β-mercaptoethanol (Sigma-Aldrich) and stored at −80°C until further processing. Total RNA was isolated with the RNeasy MicroKit according to the manufacturers’ instructions, and RNA sequencing was performed in three batches (GenomeScan). All RNA samples were polyA-enriched and then sequenced with a 150-bp paired-end read length on a NovaSeq6000 platform (Illumina).

**Table:** Specification of antibodies used for sorting of blood mononuclear cells

Antigen	Company	Clone	Dilution	Fluorochrome
CD3	eBioscience	SK7	1:250	PEcy5.5
CD4	BD Biosciences	RPA-T4	1:50	Alexa Fluor 700
CD8	BioLegend	RPA-T8	1:500	BV421
CD45RA	BioLegend	HI100	1:250	BV650
CD197/CCR7	BD Biosciences	3D12	1:10	PEcy7
LIVE/DEAD Fixable Viability Dye	eBioscience		1:1000	eFluor 780

**Table:** Specification of antibodies used for sorting of brain mononuclear cells

Antigen	Company	Clone	Dilution	Fluorochrome
CD3	eBioscience	SK7	1:250	PEcy5.5
CD4	BD Biosciences	RPA-T4	1:50	Alexa Fluor 700
CD8	BioLegend	RPA-T8	1:500	BV421
CD69	BioLegend	FN50	1:100	BV650
CD103	eBioscience	B-Ly7	1:50	PE
LIVE/DEAD Fixable Viability Dye	eBioscience		1:1000	eFluor 780

#### Flow-cytometric analyses

Single-cell suspensions were blocked with FcR Blocking Reagent and stained with cocktails of fluorochrome-conjugated antibodies in PBS supplemented with 1% fetal bovine albumin. Intracellular staining was performed using the Cytofix/Cytoperm Fixation and Permeabilization Solution kit for the detection of cytokines. Control samples included unstained, single fluorochrome-stained compensation beads (UltraComp eBeads Plus Compensation Beads), and fluorescence-minus-one (FMO) controls. Apoptotic cells were detected using Apotracker Green. Stained cells were analyzed using the BD LSRFortessa Cell Analyzer (BD Biosciences).

**Table:** Specification of antibodies used for flow-cytometric analyses

Antigen	Company	Clone	Dilution	Fluorochrome
CD3	eBioscience	SK7	1:250	PEcy5.5
CD3	eBioscience	UCHT1	1:1500	PE-CF594
CD4	BD Biosciences	RPA-T4	1:50	Alexa Fluor 700
CD4	BD Biosciences	SK3	1:250	BUV563
CD8	BD Biosciences	RPA-T8	1:250	BV786
CD8	BioLegend	RPA-T8	1:500	BV421
CD20	BioLegend	2H7	1:25	APC
CD25	BioLegend	BC96	1:25	BV421
CD39	BioLegend	A1	1:100	FITC
CD45RA	BioLegend	HI100	1:250	BV650
CD69	BioLegend	FN50	1:100	BV650
CD103	BD Biosciences	Ber-ACT8	1:100	BUV395
CD103	eBioscience	B-Ly7	1:50	PE
CD159a/NKG2A	BioLegend	S19004C	1:250	PEcy7
CD185/CXCR5	BD Biosciences	RF8B2	1:25	Alexa Fluor 488
CD192/CCR2	BioLegend	K036C2	1:25	BV510
CD195/CCR5	BioLegend	HEK/1/85a	1:100	Alexa Fluor 700
CD196/CCR6	BioLegend	G034E3	1:25	PEcy7
CD197/CCR7	BD Biosciences	150503	1:100	BUV395
CD197/CCR7	BD Biosciences	3D12	1:10	PEcy7
CD223//LAG3	BioLegend	11C3C65	1:25	BV510
CD244/2B4	BioLegend	C1.7	1:100	Alexa Fluor 700
CD279/PD-1	BD Biosciences	eBioJ105	1:10	PerCP-eFluor 710
CD366/TIM3	BioLegend	F38-2E2	1:50	BV421
Granzyme K	eBioscience	G3H69	1:50	PerCP-eFluor 710
IFNγ	BD Biosciences	25723.11	1:50	FITC
IL-2	BD Biosciences	MQ1-17H12	1:250	PEcy7
Osteopontin/OPN	R&D Systems	223126	1:25	PE
TNF	BD Biosciences	MAb11	1:50	Alexa Fluor 700
Apotracker Green	BioLegend		1:1000	FITC
LIVE/DEAD Fixable Viability Dye	eBioscience		1:1000	eFluor 780

#### Immunohistochemistry

Sections were deparaffinized, and antigen retrieval was performed in a microwave at 700 W for 10 min in Tris-EDTA buffer (pH 9.0). Endogenous peroxidase activity was blocked with 3% H_2_O_2_ and 0.2% Triton-X in Tris-buffered saline, and sections were incubated overnight at 4°C with primary antibodies.

**Table:** Specification of antibodies and procedures for immunohistochemistry

Antigen	Company	Product	Host	Clonality	Clone	Dilution	Tissue fixation	Antigen retrieval method
CD3	DAKO	A0452	rabbit	poly		1:100	FFPE	Tris-EDTA pH 9.0
OPN	Santa Cruz	sc-21742	mouse	mono	AKm2A1	1:50	FFPE	Tris-EDTA pH 9.0
TNF	Proteintech	60291-1	mouse	mono	7B8A11	1:500	FFPE	Tris-EDTA pH 9.0
GFAP	Santa Cruz	sc-6170	goat	poly		1:200	FFPE	Tris-EDTA pH 9.0

FFPE: formalin-fixed, paraffin-embedded, mono: monoclonal, poly: polyclonal.

Subsequently, sections were incubated with biotinylated anti-mouse (1:400), Alexa Fluor 488-conjugated donkey-anti-goat (1:400) and Cy3-conjugated donkey-anti-rabbit (1:400) antibodies followed by ABC (Avidin-Biotin-Complex) Kit incubation. TNF sections were then incubated for 45 min with streptavidin-Cy5 (1:600) and osteopontin (OPN) sections with biotinylated tyramide (1:10,000 in PBS with 0.001% H_2_O_2_) for 10 min and with streptavidin-Cy5 (1:600) for 45 min sequentially. For all sections, a final incubation with Hoechst for 10 min was performed. Negative controls with discard of primary antibody were included. Pictures at 40× and 63× magnification were taken using a Leica TCS SP8 confocal microscope and Application Suite X (Leica). From the same tissue blocks, OPN was stained in the same fashion as described above using the ABC kit followed by incubation with 3,30-diaminobenzidine (DAB) chromogen for 10 min. No counterstain was performed. Images were acquired using the Zeiss Axio Scan.Z1 (Carl Zeiss AG) (Figure S15).

#### *In vitro* cell stimulation

To assess intracellular cytokine production, cells were stimulated for 4 h with 10 ng/mL phorbol 12-myristate 13-acetate (PMA) plus 1 μg/mL ionomycin in the presence of 10 μg/mL brefeldin A, 5 μg/mL monensin, 2 μg/mL anti-CD28 (15E8), and 1 μg/mL anti-CD29 (TS2/16). Detection of intracellular cytokines was performed following earlier published procedures.^8^ Cells were stained with antibodies for surface markers and with LIVE/DEAD Fixable Viability Dye eFluor 780 for 30 min at 4°C. Subsequently, cells were washed, fixed, and permeabilized, using the Cytofix/Cytoperm Fixation and Permeabilization Solution kit, followed by intracellular staining with antibodies.

### Quantification and statistical analysis

#### RNAseq data preprocessing and analysis

The first dataset contained 74 samples of n = 11 brain donors with different diseases. Pooling of samples occurred in 2 instances, which were treated in the analysis as originating from an extra patient.

**Table:** Cell numbers used for RNA sequencing – First dataset

Brain donor	Diagnosis	Tissue	CD4^+^ T_EM_	CD4^+^ T_EMRA_	CD4^+^ CD69^+^	CD8^+^ T_EM_	CD8^+^ T_EMRA_	CD8^+^ CD69^+^
1	MS	CC WM			14518			55286
		OCC GM			∗238			∗∗1321
		Blood	23786	9660		14840	20118	
2	MDD	CC WM			5082			23744
		OCC GM			∗311			∗∗1616
		Blood	108388	28196		19558	39718	
3	HC	CC WM			15064			44086
		OCC GM			∗1426			8764
		Blood	9352	6132		2716	21084	
4	MSA	CC WM			6216			76804
		OCC GM			∗49			∗∗677
		Blood	56574	1904		113806	69482	
5	HC	CC WM			5670			33810
		OCC GM			∗49			∗∗711
		Blood	16170	3262		26124	24402	
6	PD	CC WM			1904			41230
		OCC GM			∗202			4186
		Blood	82166	4410		10640	70966	
7	PD	CC WM			1176			81298
		OCC GM			∗249			12726
		Blood	33936	10024		29470	17738	
8	FTD	CC WM			5180			19824
		OCC GM			∗68			∗∗855
		Blood	1788	^#^280		3710	7840	
9	AD	CC WM			5194			21000
		OCC GM			∗816			6944
		Blood	25550	2366		52346	77518	
10	HC	CC WM			9520			33026
		OCC GM			∗1621			7840
		Blood	2842	2912		24892	19026	
11	AD	CC WM			2744			38402
		OCC GM			∗249			6062
		Blood	2506	1820		1260	5124	

∗^/^∗∗Sample pooled for analysis because of low cell number. ^#^Sample excluded from analysis because of low cell number. AD: Alzheimer’s disease, CC: corpus callosum, FTD: frontotemporal dementia, GM: gray matter, HC: healthy control, MDD: major depressive disorder, MS: multiple sclerosis, MSA: multiple system atrophy, OCC: occipital cortex, PD: Parkinson’s disease, WM: white matter.

The second dataset contained 30 samples, including microglia samples, of n=6 brain donors with different diseases.

**Table:** Cell numbers used for RNA sequencing – Second dataset

Brain donor	Diagnosis	Tissue	Microglia	CD8^+^ T_EM_	CD8^+^ T_EMRA_	CD8^+^ CD69^+^
	COVID	Blood		6857	5186	
12	FTD	Sub WM	1464969			88386
		CC WM	438673			95514
		Medulla WM and GM	226975			15348
13	Narco	Sub WM	158864			22593
		CC WM	626982			67050
		Medulla WM and GM	37086			^#^3979
		Blood		73981	41493	
14	FTD	Sub WM	691510			17118
		Medulla	150022			^#^2944
15	AD	Sub WM	582000			18301
		Medulla WM and GM	212598			4824
16	FTD	Sub WM	446462			25432
		CC WM	767028			29600
		Medulla WM and GM	172396			1640

^#^Samples excluded from analysis because of low cell number. AD: Alzheimer’s disease, CC: corpus callosum, COVID: COVID-19 disease, FTD: frontotemporal dementia, GM: gray matter, M: male, Narco: narcolepsy, OCC: occipital cortex, PMD: post-mortem delay, Sub: subcortical, WM: white matter.

The third dataset contained 34 samples of n=6 MS patients.

**Table:** Cell numbers used for RNA sequencing – Third dataset

Brain donor	Diagnosis	Tissue	CD8^+^ CD69^+^	CD8^+^ CD69^+^	Lesiontype
17	MS	NAWM	19768	88032	
		NAGM	6006	27986	
		Lesional WM	882	5572	3.3
18	MS	NAWM	23422	71218	
		NAGM	952	3192	
		Lesional WM	14616	27174	3.3
19	MS	NAWM	1904	7168	
		Lesional WM		^#^155	3.2
20	MS	NAWM	1470	20496	
		NAGM		1610	
		Lesional WM		1134	3.3
21	MS	NAWM	12656	41552	
		NAGM	686	2898	
		Lesional WM	2688	10794	4
		Lesional GM	2800	13300	5.1
22	MS	NAWM	12880	41048	
		NAGM	546	1036	
		Lesional WM	15302	36736	3.3
		Lesional GM	3556	11900	5.1

^#^Sample excluded from analysis due to low cell number. GM: gray matter, MS: multiple sclerosis, NAGM: normal-appearing (perilesional) GM, NAWM: normal-appearing (perilesional) WM, WM: white matter.

Quality control was performed using FastQC (version 0.11.9). Adapter sequences were trimmed using Trimmomatic (version 0.39), and additional trimming using fastp (version 0.23.2) was performed in the second dataset. Reads were mapped to the human GRCh38 genome using HISAT2 (version 2.2.1)^57^ on default settings. Feature counts of reads mapped to genes were determined using HTSeq (first and third datasets: version 1.99.2; second dataset: version 2.0.2) with the ‘Homo_sapiens.CRCh38.105.gtf’ file (options:-m union-f bam-r name-s no-a 10-t exon-i gene_id).^64^

Resulting counts of all datasets were analyzed separately using R (version 4.2.0) and Bioconductor (version 3.15). In the first dataset, two samples were removed from analysis (one blood CD4^+^ T_EMRA_ and one WM CD4^+^ CD69^+^ T cells). The blood CD4^+^ T_EMRA_-cell sample was removed due to small library size (2824565). The WM CD4^+^ CD69^+^ T-cell sample was removed due to a low cell number input (1176) in combination with an unusually low normalization factor (0.74) and low expression of sample hallmark gene *CD4*. In the second dataset, three samples were removed from analysis based on initial PCA. Two samples (one CD8^+^ medulla T cells and one CD8^+^ subcortical WM T cells) had an aberrant library prep, and one sample (CD8^+^ medulla T cells) contained the lowest input cell number. Plots were not corrected for effects relating to input, as this was related to experimental groups. Initial data exploration was performed using both microglia and T-cell data, afterwards, T-cell data were subsetted, normalized again and used for further analysis. Based on an initial principal component analysis (PCA), one sample was also removed from the third dataset. This sample (CD4^+^ normal-appearing GM T cells) contained a low cell number (546) used for sequencing analysis. Genes with more than 2 counts in a minimum of 4 (first dataset) or 2 (second and third datasets) samples were retained. Count data were transformed to log2-counts per million (logCPM), normalized using the trimmed mean of M-values (TMM) method (edgeR package, version 3.37.4),^59^ and precision weighted using voom (limma package, version 3.51.8).^65^ The remaining genes (first dataset 1: 23,712, second dataset: 22,632, and third dataset: 24,077) were reannotated with the biomaRt package (version 2.51.4),^57^ using Ensembl (version 106). PCA was performed on the logCPM values of the 500 most variable genes to distinguish sources of variation using the PCAtools package (version 2.8.0). In the first and third datasets, the first principal component was strongly correlated with the log10-transformed cell number counts. Therefore, principal component plots were made after correction for this covariate and donor variation, using the function removeBatchEffect (limma package). Plots in which the first and third datasets are shown were generated using a merged dataset, which was obtained by combining the raw counts and applying filtering and normalization as described above. Gene expression values were corrected for cell number and donor effects using the limma package.

Differential expression was assessed using an empirical Bayes moderated t-test within limma’s linear model framework^71^ including donor and log10-transformed cell number counts as covariates [Y = ∼0 + experimental condition + donor + log_10_ (cell number)]. In the second dataset, differential expression was only assessed of T cells. Resulting p values were corrected for multiple testing using the Benjamini-Hochberg false discovery rate (FDR). Genes with FDR <0.05 were considered significantly differentially expressed. Expression of *SPP1* by microglia and T cells was quantified on the same data as the original exploration and tested using a Wilcoxon signed-rank test in GraphPad Prism. Results were visualized with the ggplot2 package (version 3.3.5).^62^

Competitive gene set enrichment analysis was performed with CAMERA^72^ with preset value of 0.01 for the inter-gene correlation using the Hallmark, C1, C2, C3, C5, C6, and C7 gene set collections retrieved from the Molecular Signatures Database (MSigDB, version 7.0; https://www.gsea-msigdb.org/gsea/msigdb/index.jsp),^68^ T_RM_-cell core signature^13^ and MS risk allele gene sets^20^ were added manually. CAMERA results were visualized using Cytoscape (version 3.9.0) with the EnrichmentMap (version 3.3.3) plugin^58^^,^^60^ with a cutoff of FDR <0.1 and a minimum of 5 gene sets per network. The less conservative criterion was chosen to create a less sparse network. Networks were portioned into modules and annotated using the AutoAnnotate (version 1.3.5) plugin and manual curation.

#### Flow cytometry

Flow-cytometric data were analyzed using FlowJo software. Graphs were visualized using GraphPad Prism.

#### Immunohistochemistry

For immunohistochemical quantification of OPN^+^ CD3^+^ cells, images of CD3^+^ cell clusters were taken at 40× magnification. Data were then analyzed using QuPath 0.2.3.^66^ Cell detection was run using StarDist.^73^ CD3^+^ cells were classified using a QuPath-integrated random trees object classifier. OPN^+^ and TNF^+^ cells were classified using a single measurement classifier with a threshold mean cell intensity of 2.5 (OPN, control tissue) or 6.0 (OPN, MS tissue) and 12.5 (TNF, MS tissue), respectively.

Regions-of-interest were annotated with holes and tissue inconsistencies excluded. OPN-positive area was established through a pixel thresholder including a Laplacian of Gaussian prefilter. Statistical analyses of all image quantifications were performed in R (version 4.0.2) using a mixed model approach including donor as a random factor through the ‘lme4’ (version 1.1-29) and ‘emmeans’ (version 1.8.0) packages. Graphs were visualized using GraphPad Prism.

## Data Availability

• The RNA-sequencing dataset have been deposited in the Gene Expression Omnibus (GEO) database (https://www.ncbi.nlm.nih.gov) under accession number GSE216030. • Sequencing results are also available for browsing at the Quickomics platform. • First dataset, circulating vs brain-resident T cells: http://quickomics.bxgenomics.com/?serverfile=Quickomics_BrainTcell_Circulating. • Second dataset, T cells from different brain regions: http://quickomics.bxgenomics.com/?serverfile=Quickomics_BrainTcell_Location. • Third dataset, T cells from MS normal-appearing vs lesional tissues: http://quickomics.bxgenomics.com/?serverfile=Quickomics_BrainTcell_MS. • This paper does not report original code. • Any additional information required to reanalyze the data reported in this paper is available from the lead contact upon request.

## Supplemental information

Document S1. Figures S1–S15

Data S1. DE gene analysis for CD8^+^ and CD4^+^ T cells from WM, GM, and paired peripheral blood of n = 11 brain donors, related to Figure 2B

Data S2. Gene set enrichment analysis results of T cells from different locations, related to Figures 4E and S7

Data S3. DE gene analysis for CD8^+^ T cells from corpus callosum WM, subcortical WM, and medulla WM/GM of n = 3–5 brain donors, related to Figure 4F

Data S4. DE gene analysis for CD8^+^ and CD4^+^ T cells from MS normal-appearing and lesional WM and GM of n = 6 brain donors, related to Figure 5E

Data S5. Gene set enrichment analysis results of MS donors, related to Figure S10A

Data S6. Gene set enrichment analysis results of matched blood and brain samples, related to Figures S10B and S10C
