# Supplementary material for: Osteopontin associates with brain TRM-cell transcriptome and compartmentalization in donors with and without multiple sclerosis
Source: iScience. 2022 Dec 9;26(1):105785. doi: 10.1016/j.isci.2022.105785 (PMC9804143; doi:10.1016/j.isci.2022.105785)
Supplement: Document S1. Figures S1–S15 [file mmc1.pdf]

iScience, Volume *26*

Supplemental information

Osteopontin associates with brain T_{RM}-cell transcriptome and compartmentalization in donors with and without multiple sclerosis

Cheng-Chih Hsiao, Hendrik J. Engelenburg, Aldo Jongejan, Jing Zhu, Baohong Zhang, Michael Mingueneau, Perry D. Moerland, Inge Huitinga, Joost Smolders, and Jörg Hamann

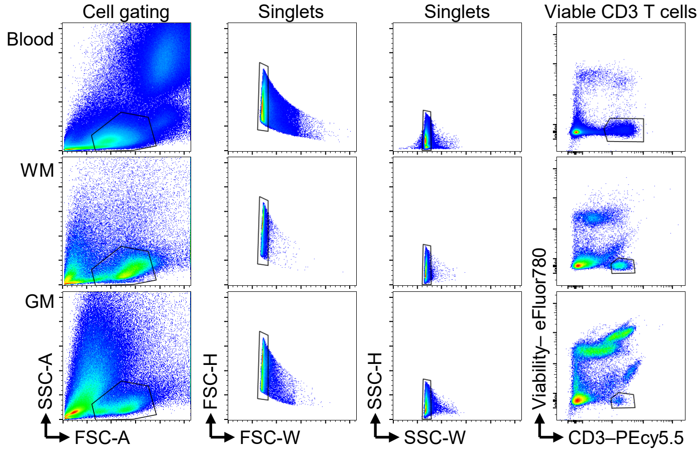

Figure S1 related to Figure 1A: Flow cytometry gating strategy. Representative dot plots showing the gating strategy used for cell sorting and flow cytometry with from left to right cell gating by forward scatter (FCS) and sideward scatter (SSC), FCS-width/height duplet exclusion, SSC-width/height duplet exclusion, gating of viable CD3$^+$ T cells. The rows from top to bottom represent paired peripheral blood, WM, and GM.

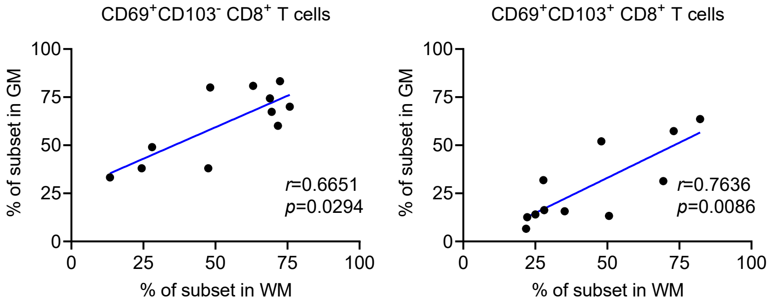

Figure S2 related to Figure 1B: Correlation of $CD69^{+}CD103^{+/-}$ $CD8^{+}$ T cells between WM and GM, determined by flow cytometry. The strong correlation of $CD69^{+}CD103^{-}$ and $CD69^{+}CD103^{+}$ $CD8^{+}$ T_{RM}-cell proportions between WM and GM (Spearman r=0.6651, p=0.0294 and Spearman r=0.7636, p=0.0086, respectively).

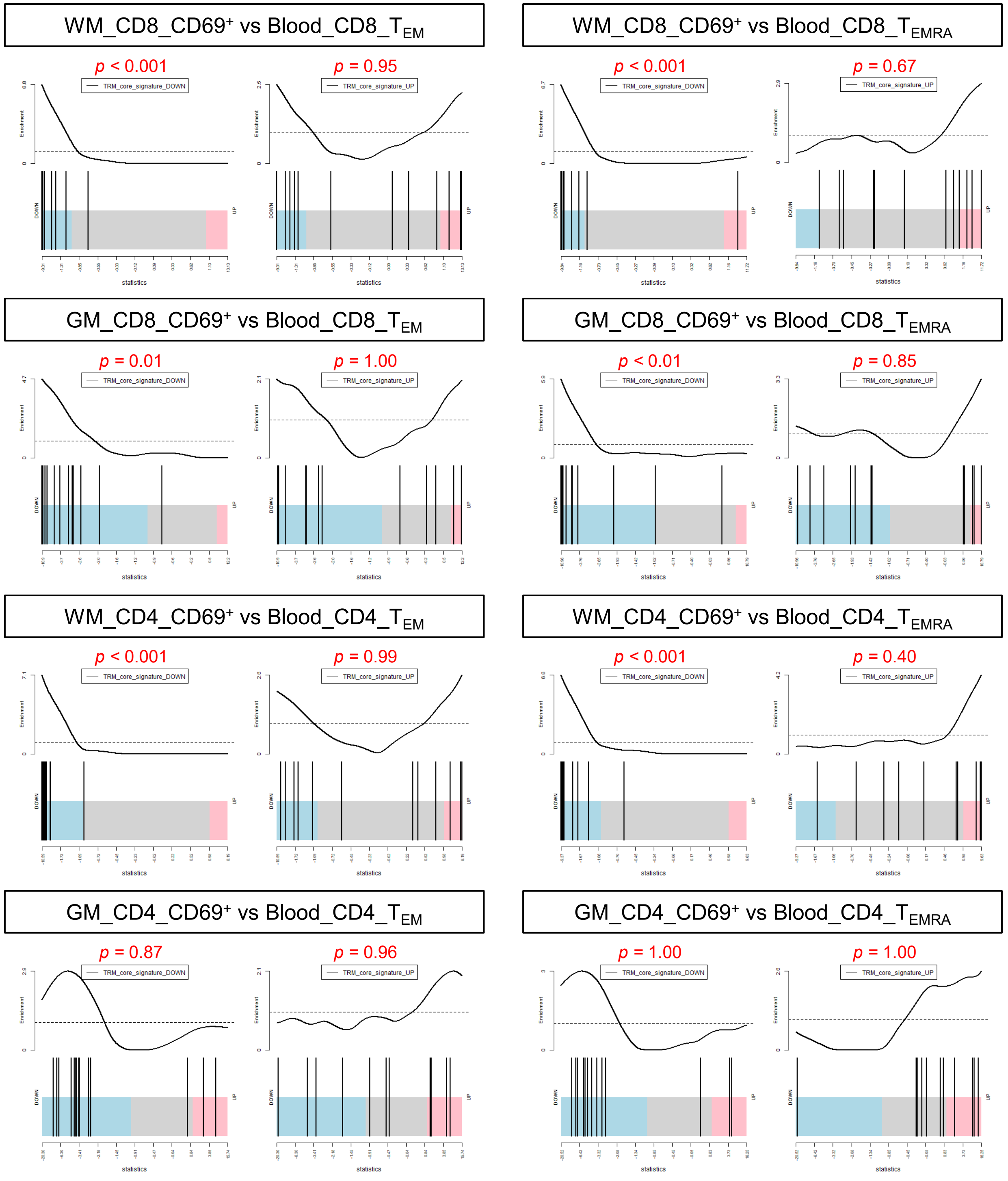

Figure S3 related to Figure 1E: Competitive gene set enrichment analysis (CAMERA) of the T_{RM}-cell associated gene set in the first dataset. Genes are ranked by differential expression for the indicated comparison with bars corresponding to genes associated with tissue residency. The black curve indicates relative enrichment.

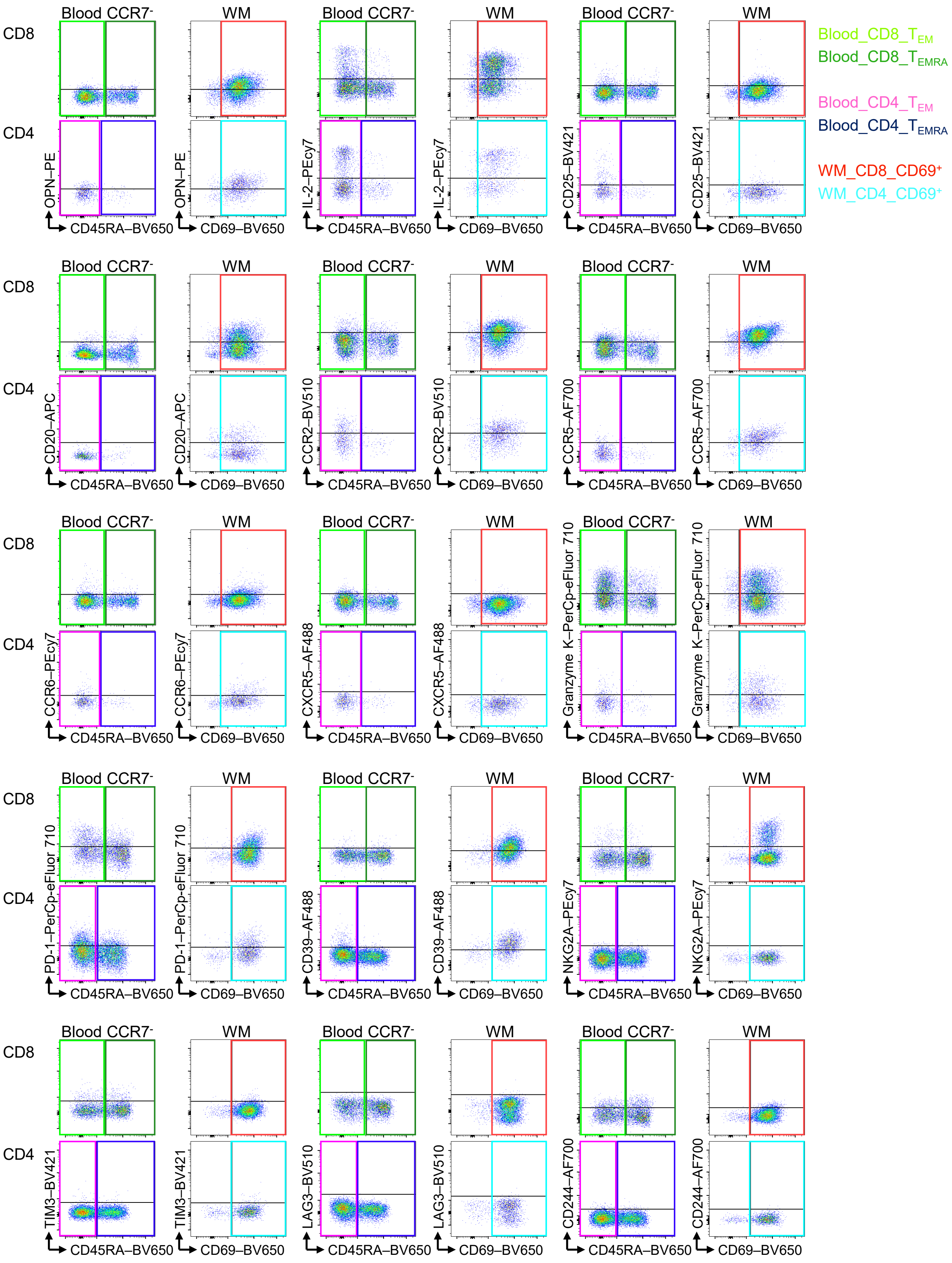

Figure S4 related to Figure 2D, 6B, and 6D: Representative dot plots of CD8⁺ and CD4⁺ blood and WM T cells stained for surface and intracellular markers by flow cytometry. Markers shown here are OPN, IL-2, CD25, CD20, CCR2, CCR5, CCR6, CXCR5, granzyme K, PD-1, CD39, NKG2A, TIM3, LAG3, and CD244.

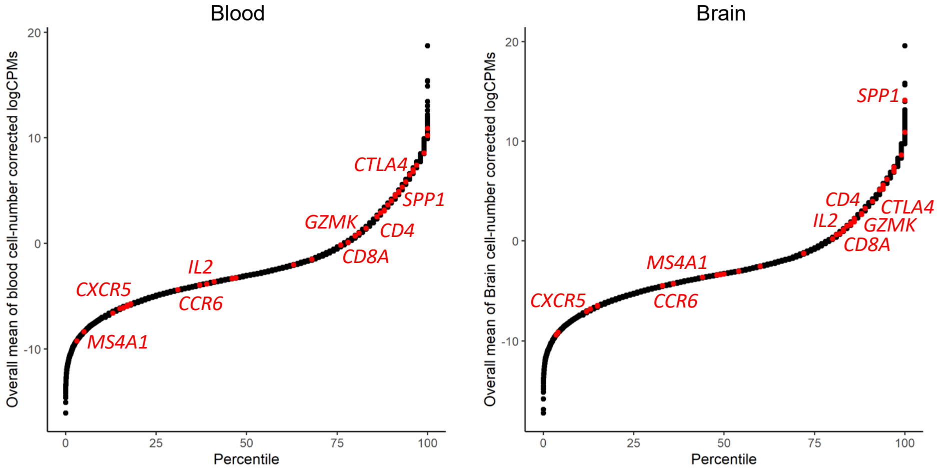

Figure S5 related to Figure 2: Relative abundance of genes of interest in the first dataset. Average-log_2CPM (corrected for cell number using limma removeBatchEffect function) and percentile of expression level in all groups of the first dataset donors, separated by blood and brain derived cells.

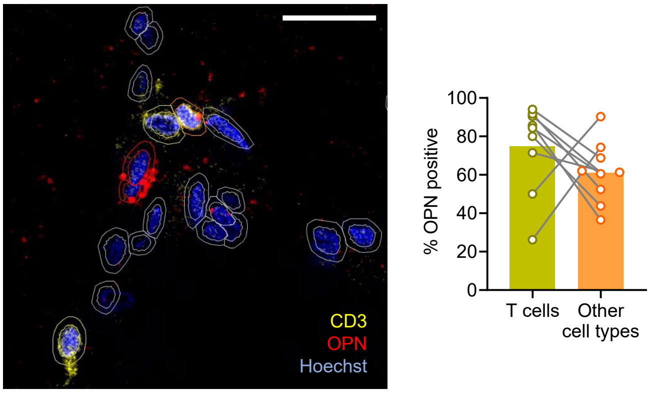

Figure S6 related to Figure 3B: Quantification of OPN$^+$-expressing T cells. Left panel: Example of cell classification of an immunofluorescent staining. Shown are the cell detections with corresponding expansion. 40 x magnification; scale bar = 40 µm. Right panel: Ratio of CD3$^+$ T cells expressing or being in contact with OPN, compared to other cell types.

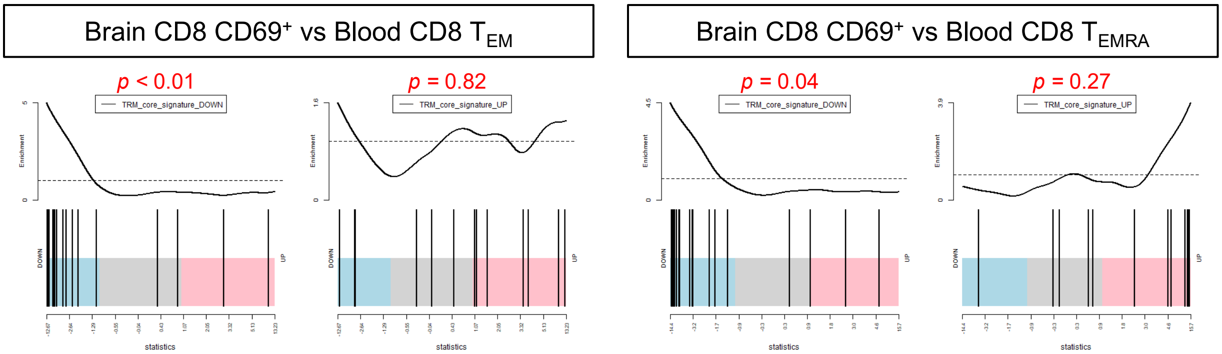

Figure S7 related to Figure 4E: Competitive gene set enrichment analysis (CAMERA) of the T_{RM}-cell associated gene set in the second dataset. Genes are ranked by differential expression for the indicated comparison with bars corresponding to genes associated with tissue residency. The black curve indicates relative enrichment.

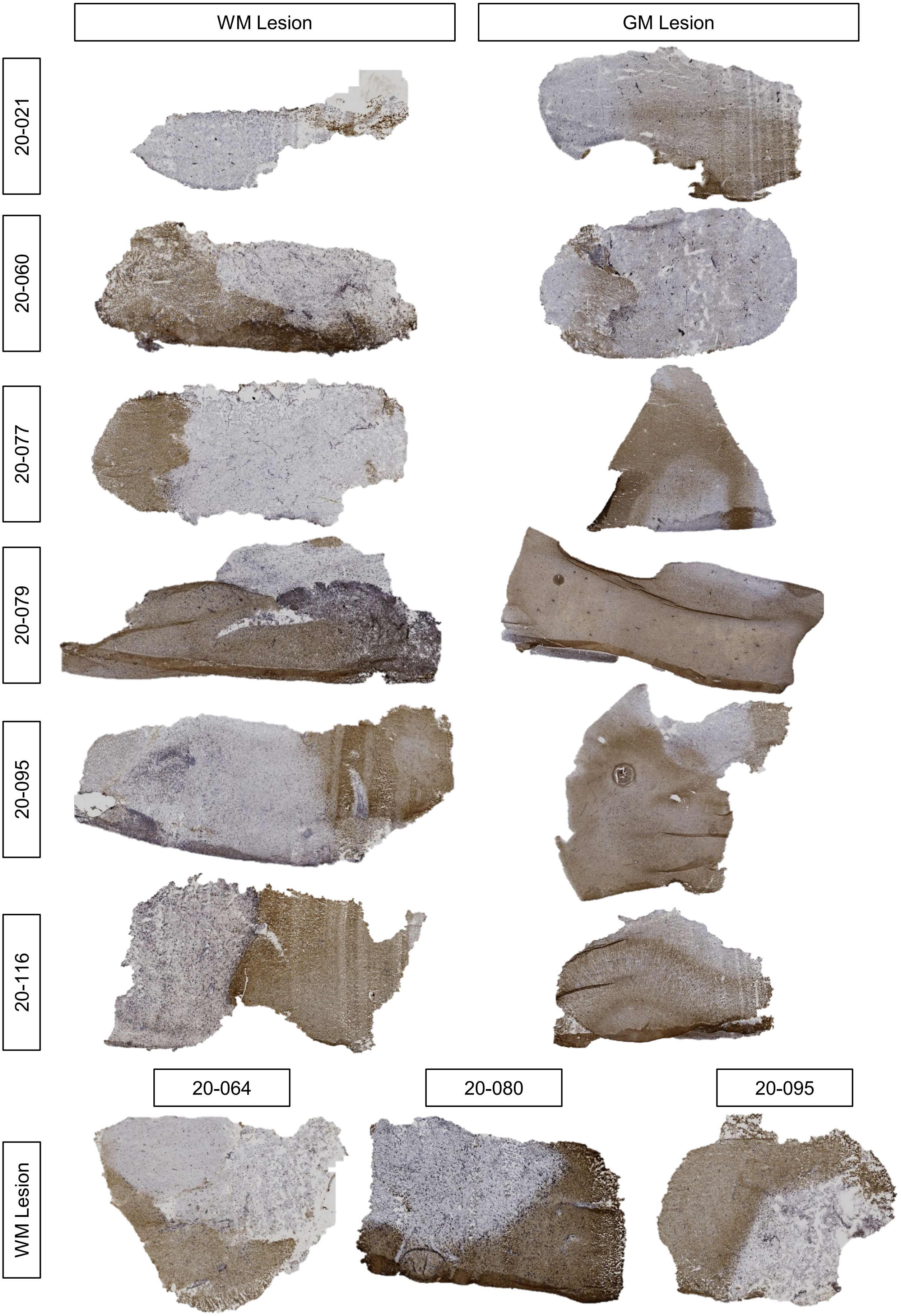

Figure S8 related to Figure 5A: Immunohistochemical staining of the isolated WM and GM lesions processed for the third dataset. In black, human leukocyte antigen (HLA-DP/DQ/DR) and in brown, human proteolipid protein (PLP) is visualized.

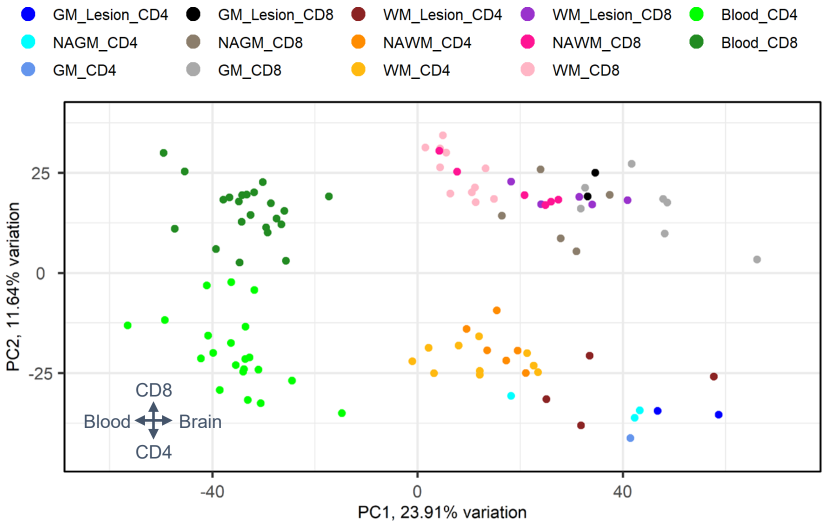

Figure S9 related to Figure 5B: Combined PCA of the first and third datasets. No batch correction between batches has been performed.

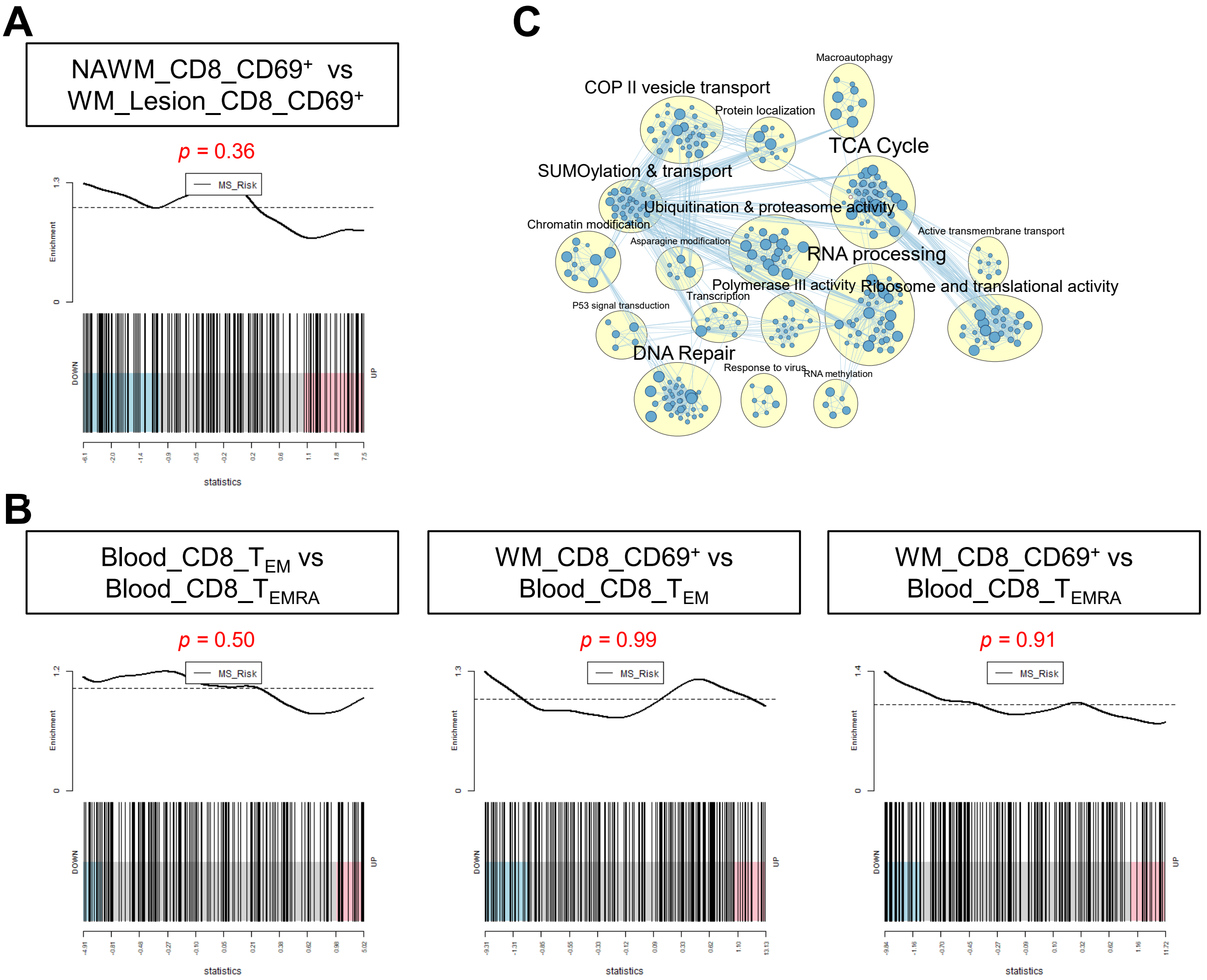

Figure S10 related to Figure 5: Summary of CAMERA gene set enrichment analysis results in CD8$^+$ T$_{RM}$ cells from MS normal-appearing and lesional WM. (**A**, **C**) Genes are ranked by differential expression for the indicated comparison with bars corresponding to genes associated with MS risk alleles. The black curve indicates relative enrichment. (**B**) Network visualization of the CAMERA gene set enrichment analysis results. Each node corresponds to a gene set with FDR <0.1, and a network is formed based on overlap between gene sets as indicated by edges connecting pairs of gene sets. Only modules with more than 5 nodes are shown. Modules with limited relevance to T cells are not shown. Blue nodes were enriched in WM lesions and a single red node was upregulated in NAWM.

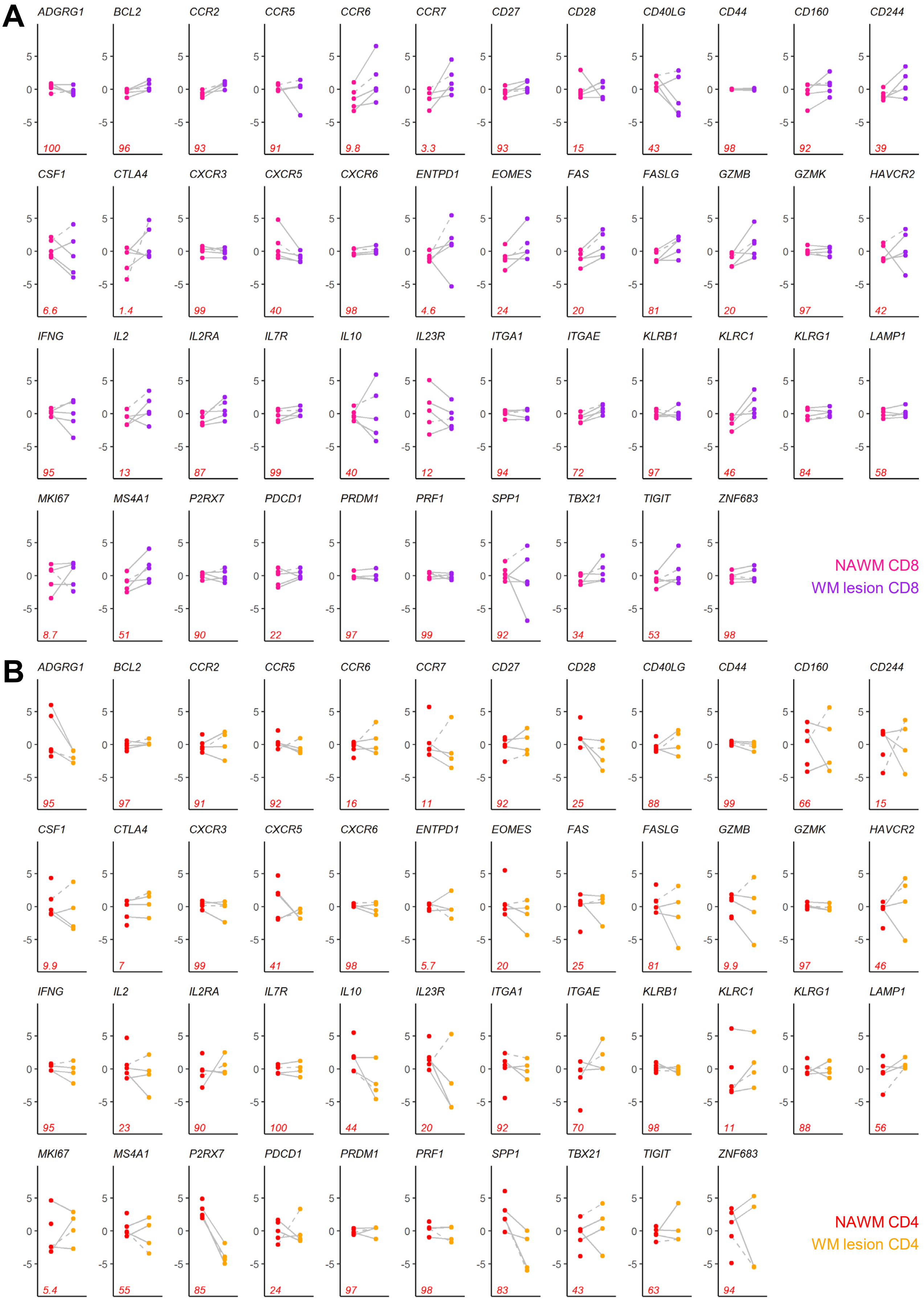

Figure S11 related to Figure 5: Comparison of gene expression in MS normal-appearing and lesional WM in the third dataset. For discovery of possible trends, z-scored log_2CPM values of T-cell relevant genes in (**A**) $CD8^+$ and (**B**) $CD4^+$ T cells are plotted. Numbers in red signify the percentile of gene expression across samples.

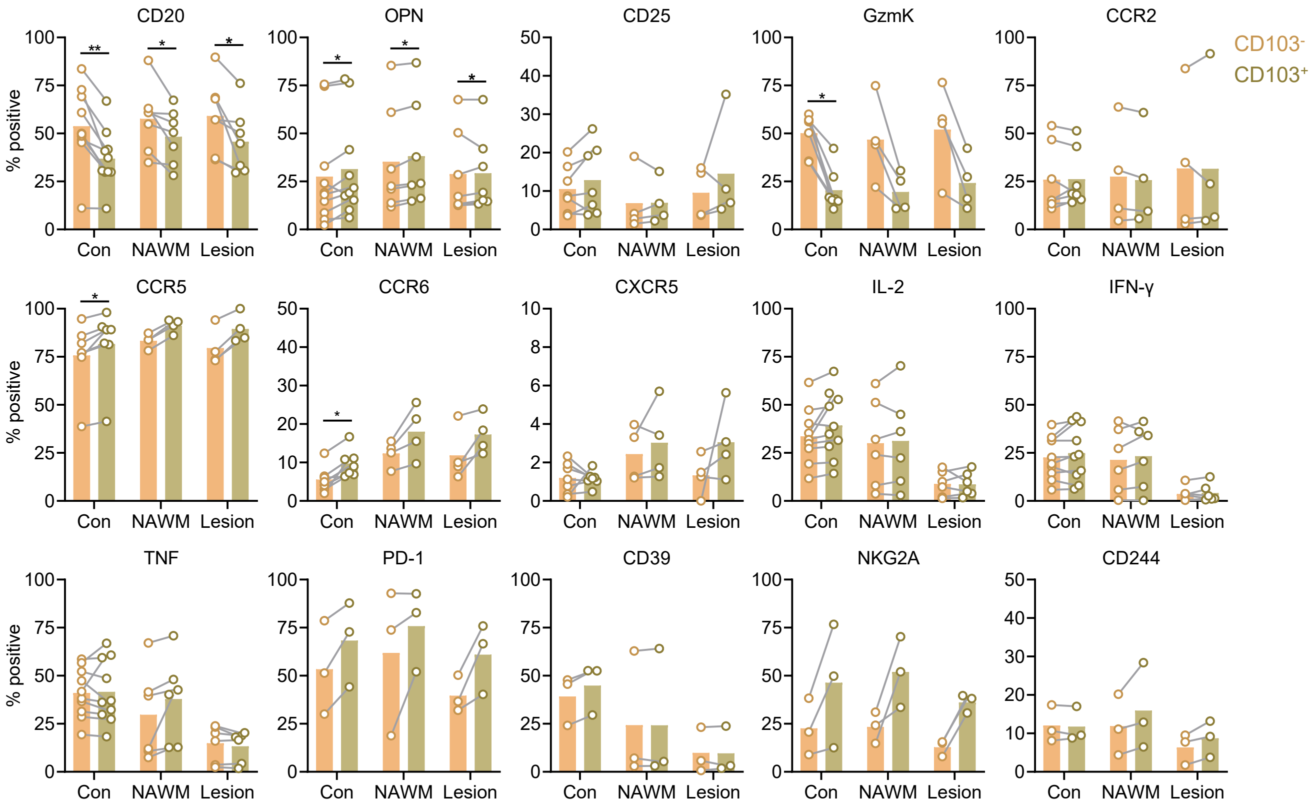

Figure S12 related to Figure 6: Selected CD8$^+$ T-cell proteins stratified for CD103 expression. Shown is the percentage of CD8$^+$ cells positive for multiple proteins of interest in control and MS normal-appearing and lesional WM, stratified for CD103 expression. Wilcoxon signed-rank test was used, and *p*-value is shown in the plots. ** $p < 0.05$; ** $p < 0.01$.*

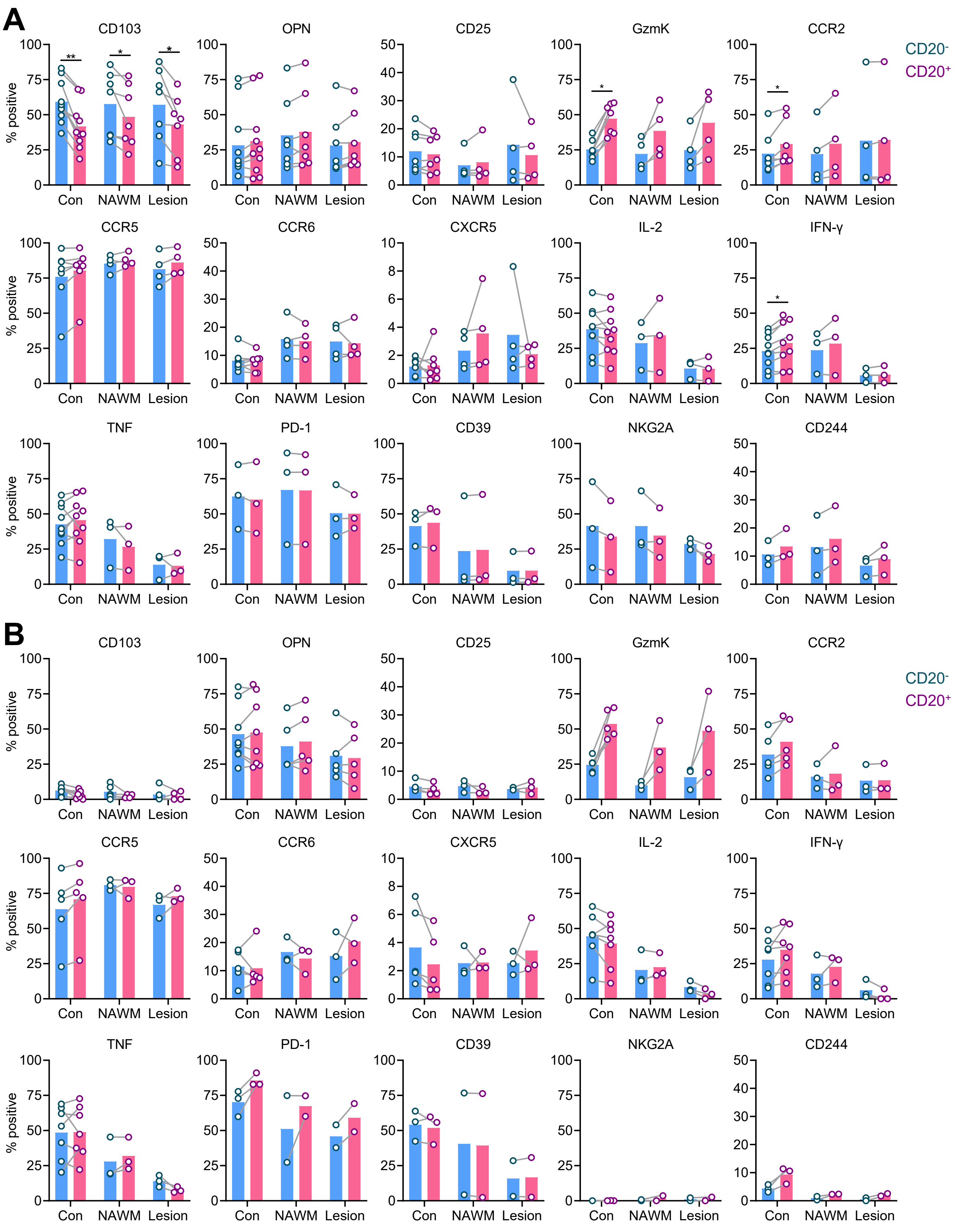

Figure S13 related to Figure 6: Selected $CD8^+$ and $CD4^+$ T-cell proteins stratified for CD20 expression. Shown is the percentage of (**A**) $CD8^+$ or (**B**) $CD4^+$ cells positive for multiple proteins of interest in control and MS normal-appearing and lesional WM, stratified for CD20 expression. Wilcoxon signed-rank test was used, and *p*-value is shown in the plots. ** $p < 0.05$; ** $p < 0.01$.*

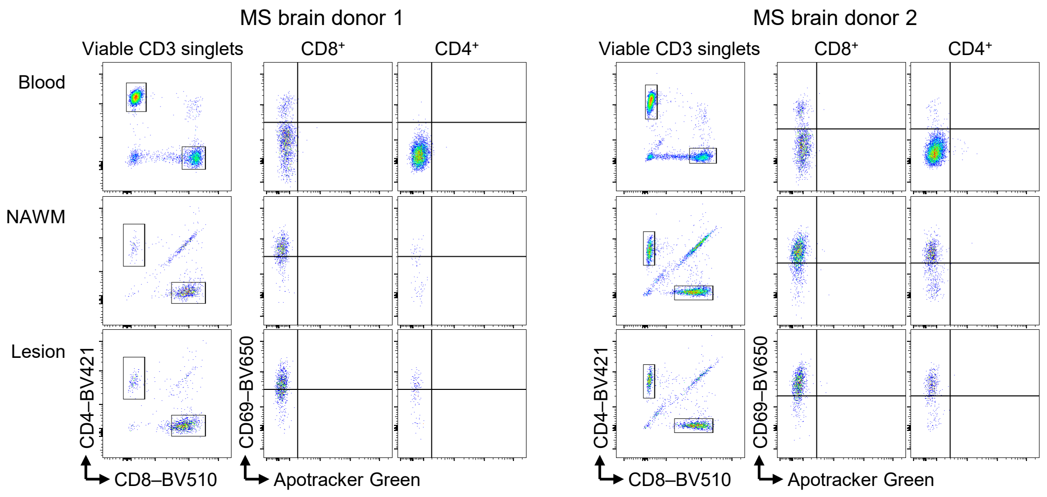

Figure S14 related to Figure 6C: No induction of apoptotic CD8⁺ and CD4⁺ T cells in MS lesion. Dot plot showing apoptotic CD8⁺ and CD4⁺ T cells from n=2 matched blood and brain by Apotracker Green stainings.

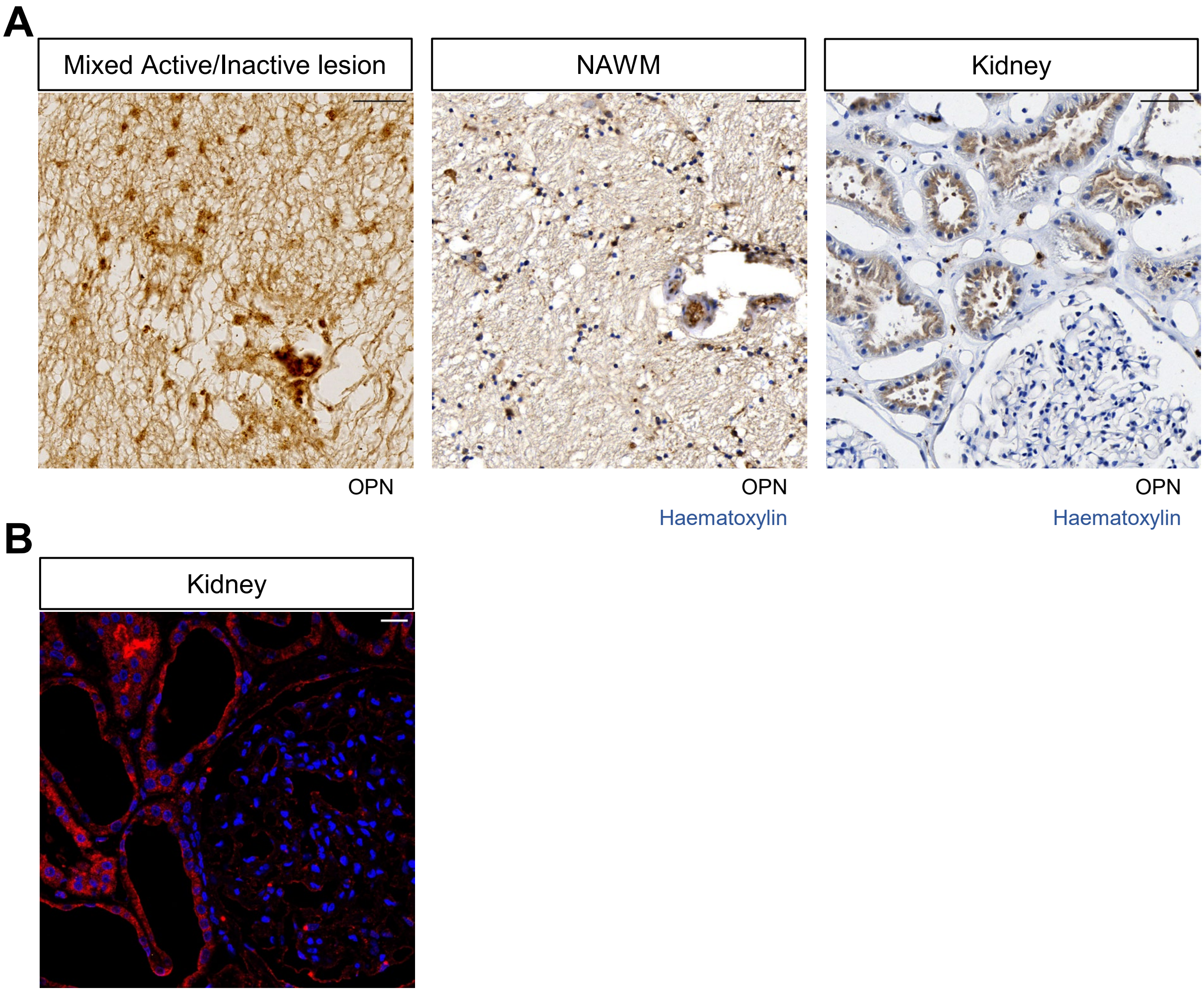

Figure S15 related to Figure 7B: OPN Immunohistochemistry. (**A**) Examples of immunohistochemistry staining of OPN in lesions, as used for positive area quantification, normal-appearing WM, with hematoxylin to visualize cell association, and kidney, as positive control with only staining of the tubuli. (**B**) Immunofluorescence of OPN in kidney as a positive control with signal most prominent in the tubuli. Scale bars indicate 50 µm.
